# Synthesis of New Volatile Derivatives of Biogenic Amines, Carbamates for Analytical Applications

**DOI:** 10.3390/ma19030575

**Published:** 2026-02-02

**Authors:** Kamil Brzuzy, Aneta Jastrzębska, Anna Kmieciak, Jacek Ścianowski, Tadeusz Muzioł, Damian Gorczyca, Marek P. Krzemiński

**Affiliations:** 1 Department of Analytical Chemistry and Applied Spectroscopy, Faculty of Chemistry, Nicolaus Copernicus University in Toruń, 7 Gagarin Str., 87-100 Toruń, Poland; kamil.brzuzy@freshinset.pl; 2 Fresh Inset, Wileńska 4/A017 Str., 87-100 Toruń, Poland; 3 Department of Organic Chemistry, Faculty of Chemistry, Nicolaus Copernicus University in Toruń, 7 Gagarin Str., 87-100 Toruń, Poland; akmieciak@umk.pl (A.K.); jsch@umk.pl (J.Ś.); 4 Department of Inorganic and Coordination Chemistry, Faculty of Chemistry, Nicolaus Copernicus University in Toruń, 7 Gagarin Str., 87-100 Toruń, Poland; tmuziol@chem.umk.pl; 5 Faculty of Medicine, Vizja University, Okopowa 59 Str., 01-043 Warsaw, Poland; damiang@lymelabpharma.com; 6 LymeLab Pharma Sp. z o.o. Sp. k., Kochanowskiego 49A Str., 01-864 Warsaw, Poland

**Keywords:** biogenic amines, gas chromatography, derivatization, ethyl chloroformate, 2,2,2-trifluoroethyl chloroformate, food samples

## Abstract

In this study, a comprehensive derivatization strategy for biogenic amines based on the formation of volatile carbamate derivatives using 2,2,2-trifluoroethyl chloroformate (TFECF) was successfully developed and evaluated. A series of biogenic amine derivatives was obtained in excellent yields (94–99%) and structurally confirmed using NMR, MS, and crystal structure analysis. The reagent demonstrated high reactivity toward primary and secondary amines, providing derivatives of excellent purity and satisfactory volatility. The applicability of the proposed procedure to real food matrices was demonstrated using GC-MS. The obtained results were compared with the corresponding ethyl chloroformate (ECF) derivatives. TFECF derivatives exhibited significantly improved volatility, reflected in shorter retention times and enhanced analytical performance.

## 1. Introduction

**Figure 1:**
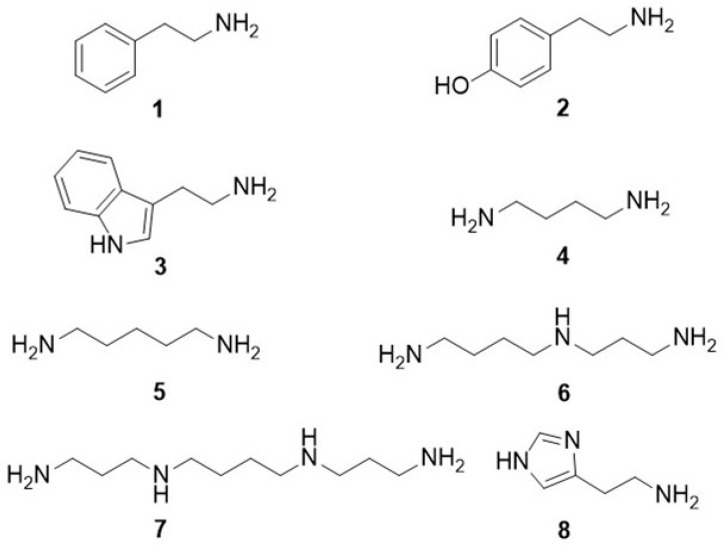
Structural formulas of BAs occurring in food: phenylethylamine (**1**), tyramine (**2**), tryptamine (**3**), putrescine (**4**), cadaverine (**5**), spermidine (**6**), spermine (**7**), and histamine (**8**).

Biogenic amines (BAs) constitute a broad group of low-molecular-weight organic compounds containing nitrogen, characterized by diverse structures and biologically active properties. Some of them, such as dopamine, norepinephrine, epinephrine, and serotonin, are involved in the control and regulation of fundamental physiological functions and behaviors. At the same time, these compounds are associated with the pathology of various neurological disorders, and their presence or deficiency may serve as an indicator of disease development [1]. Moreover, among the metabolites in the human body, biogenic amines and their precursors—amino acids—play central roles as substrates in organ metabolism [2]. Additionally, polyamines are essential for cell growth and differentiation and are widely recognized as cancer biomarkers [3]. Some BAs are present in various food products rich in animal protein or obtained through fermentation processes [4]. The most common BAs found in foods are phenylethylamine (**1**), tyramine (**2**), tryptamine (**3**), putrescine (**4**), cadaverine (**5**), spermidine (**6**), spermine (**7**), and histamine (**8**) (Figure 1) [5].

Since the consumption of foods containing high concentrations of biogenic amines (BAs) may lead to various adverse health effects, regulatory authorities worldwide, such as the European Food Safety Authority (EFSA), the World Health Organization (WHO), and the Food and Drug Administration (FDA) have established food-specific regulations and maximum permissible levels for selected biogenic amines (primarily histamine), rather than general limits applicable to all food products [1,6,7]. Moreover, the content of biogenic amines has become an important quality indicator in many food products [8,9].

The presence of these compounds in food can be detected by identifying amino acid decarboxylase microorganisms capable of producing them. The instrumental methods enabling the direct quantitative determination of biogenic amines are far more widely employed. However, analysis of these compounds is complex due to their diverse structures and low concentrations, the complexity of the food samples, and the presence of interfering compounds. Therefore, the derivatization step is often necessary to address most analytical challenges and to enhance detection sensitivity. Despite numerous methods for determining BAs in food and biological fluids, HPLC, GC, and CE are the routine techniques used in most BAs’ analysis [5,9,10,11]. Moreover, the inclusion of a derivatization step enhances the detectability and stability of the analyzed compounds, thereby significantly improving analytical performance [12].

Derivatization requires the presence of a reactive functional group in the target compounds and the use of a suitably chosen reagent that improves detection sensitivity through the incorporation of fluorophoric or chromophoric moieties. Furthermore, the reduction in BAs polarity improves their extraction and chromatographic separation. On the other hand, derivatization can be considered an additional step in sample preparation that slows down the entire analysis, introduces additional impurities, reduces accuracy, and introduces uncertainty regarding the stability of the derivatives. Therefore, adherence to the principles of green analytical chemistry is essential, including the use of low levels of derivatizing reagents, the minimization of time and energy consumption, and the use of less toxic reagents [12].

In the case of the GC technique, derivatization is a mandatory step that contributes to the volatility of the tested compounds and reduces polarity, which improves the retention of compounds in the chromatographic column. According to Munir and Badri [13], derivatizing reagents commonly used in GC are divided into three main groups: alkylating, acylating, and silylating agents. Among them, silylation—despite certain drawbacks such as the need for heating and the reagent’s susceptibility to moisture, which may lead to its deactivation—is the most frequently described in the literature [13].

Given that many derivatization procedures have become broadly applicable in recent years, the primary aim of this study was to optimize the formation of volatile carbamate derivatives of biogenic amines, enabling their reliable determination by GC using a widely employed flame ionization detector (FID). These reactions can be readily performed in an aqueous alkaline medium in the presence of a catalyst; however, their yields are usually low [14].

Therefore, the objective of this study was to develop and comprehensively evaluate a derivatization strategy for BAs based on the formation of volatile carbamate derivatives using 2,2,2-trifluoroethyl chloroformate (TFECF). Particular emphasis was placed on confirming the molecular architecture of the synthesized derivatives through high-resolution mass spectrometry and single-crystal X-ray diffraction. TFECF has been described in two publications so far. In the first, 2,2,2-trifluoroethyl chloroformate was used to derivatize amino acids [15]. The second publication reported the analysis of 1,6-hexamethylenediamine in urine using TFECF [16]. Moreover, TFECF has not yet been applied in analytical protocols for the determination of BAs in food matrices and was explored here as a potentially more volatile and analytically advantageous analog of ethyl chloroformate (ECF). Finally, the analytical applicability of the proposed approach was demonstrated by quantifying phenylethylamine in selected fermented vegetable juices, highlighting the method’s suitability for real-sample analysis using the GC-MS technique.

## 2. Materials and Methods

### 2.1. Reagents

Analytical grade: 2-phenylethylamine, tyramine, tryptamine, putrescine, cadaverine, spermidine, spermine, histamine, chloroform-*d* (CDCl_3_), 2,2,2-trifluoroethanol (TFE), triphosgene (bis(trichloromethyl) carbonate (BTC)), ethyl chloroformate (ECF) were purchased from Merck KGaA (Darmstadt, Germany), whereas methanol for HPLC (MeOH), tetrahydrofuran (THF), sodium hydroxide, diethyl ether (Et_2_O), dichloromethane (DCM), ethyl acetate (EtOAc), triethylamine (Et_3_N), and magnesium sulfate (anhydrous) were purchased from Alchem (Poland). Methanol LC-MS was acquired from ChemSolute (Th.Geyer Gmbh & Co Kg, Renningen, Germany), formic acid for LC-MS was acquired from LiChropur, Merck (Merck KGaA, Darmstadt, Germany). Deionized water was obtained from a Rephile water purification system (ephiLe Bioscience, Ltd., Acton, MA, USA).

### 2.2. Procedures

#### 2.2.1. 2,2,2-Trifluoroethyl Chloroformate (TFECF)

Triphosgene (6.0 g, 20 mmol) was dissolved in DCM (60 mL) in a 100 mL round-bottom flask equipped with a magnetic stir bar and connected to an oil bubbler. 2,2,2-Trifluoroethanol (4.4 mL, 60 mmol) was then slowly added via syringe, followed by the addition of Et_3_N (8.4 mL, 60 mmol). The reaction mixture was stirred at room temperature overnight and poured into Et_2_O (100 mL). The precipitated triethylammonium chloride was removed by filtration under reduced pressure. The crude TFECF was purified by fractional distillation using a Vigreux column. The fraction boiling at 67–69 °C was collected. The purity of the obtained product was confirmed by NMR study.

^1^H NMR (400 MHz, CDCl_3_) *δ* [ppm]: 4.66 (q, J = 7.92 Hz, 2 H); ^13^C NMR (101 MHz, CDCl_3_) *δ* [ppm]: 65.43 (q, J = 37.76 Hz, OCH_2_); 121.75 (q, J = 277.40 Hz, CF_3_); 150.45 (C=O); ^19^F NMR (376 MHz, CDCl_3_) *δ* [ppm]: −74.75.

#### 2.2.2. General Procedure for the Synthesis of the BA Carbamates

In a typical procedure for TFECF derivatives, the BA (1 mmol) was placed in a 25 mL round-bottom flask and combined with EtOAc, water, and 2 M NaOH in amounts adjusted to the number of amino groups. For monoamines, EtOAc (0.70 mL), water (0.85 mL), and NaOH (1.1 equiv, 0.55 mL 2 M, 1.1 mmol) were used; for diamines, EtOAc (0.70 mL), water (0.30 mL), and NaOH (2.2 equiv, 1.1 mL 2 M, 2.2 mmol); for triamine—spermidine, EtOAc (0.85 mL) and NaOH (3.3 equiv, 1.65 mL 2 M, 3.3 mmol) (no added water); and for tetraamine spermine, EtOAc (1.10 mL) and NaOH (4.4 equiv, 2.2 mL 2 M, 4.4 mmol) (no added water). The mixture was stirred and cooled to 5–10 °C in an ice bath, and then TFECF (1.1–4.4 equiv, depending on the amine functionality) was added dropwise. The reaction was maintained at a temperature of 5–10 °C for 15 min and then stirred for an additional 2 h at room temperature. The product was extracted with EtOAc (3 × 10 mL), dried over MgSO_4_, filtered, and concentrated under reduced pressure. Crude derivatives were purified by silica gel column chromatography (Table S1).

The same procedure was applied for ECF derivatives, whereas EtOAc was replaced by Et_2_O.

### 2.3. Analysis Data—^1^H, ^13^C, ^19^F NMR, and MS Studies and Crystal Structure Determination

NMR spectra were recorded on a Bruker Avance III 400 MHz spectrometer at 400 MHz for ^1^H and 101 MHz for ^13^C frequency resonances, and 376 MHz for ^19^F at 298 ± 1 K, in CDCl_3_-*d*. Chemical shifts are reported in ppm using the residual CDCl_3_ peaks as references: δ 7.26 for ^1^H NMR and relative to the central δ 77.00 resonance signal for ^13^ C NMR. Spin–spin coupling constants (J) are in Hz. The HRMS experiments were performed using a Nexera 2 LC-3AD (Shimadzu Corporation, Japan) interfaced with an ion trap IT-TOF (Shimadzu Corporation, Japan) with electrospray ionization (ESI). The mass spectrometer was operated in positive ion mode using an ionization voltage of 1,55 kV. The ion transfer capillary temperature was 200 °C. Nitrogen sheath gas and auxiliary gas were set at 30 and 10 arbitrary units, respectively. The instrument was calibrated using a solution containing NaTFA in acetonitrile, as per the manufacturer’s instructions. The calibration was performed before analysis. Data were acquired with a *m*/*z* range of 150–800 and a drying gas pressure of 108 kPa. The mass spectrometer was operated in LC-MS Solution software (LCMSsolutionVer 3) (Shimadzu Corporation, Kyoto, Japan).

The LCMS-IT-TOF ion trap generated product ions at a normalized collision energy with an ion accumulation time of 10 msec. Standard stock solutions of each compound were prepared at a concentration of 1 mg/mL in LC-MS methanol. Working solutions (100 ng/mL) were prepared by diluting the stock solution 100-fold with LC-MS-grade methanol. LC separation was performed by direct injection using isocratic elution at a constant flow rate of 0.4 mL/min. The LC eluents were solvent A (deionized water with 0.1% HCOOH) and solvent B (MeOH with 0.1% HCOOH): 50% A and 50% B in isocratic elution.

The diffraction data of **1.1**, **1.2**, **4.1**, **4.2**, **5.1**, and **5.2** were collected at 100 K for the single crystal on Rigaku XtaLAB Synergy (Dualflex) diffractometer with HyPix detector with monochromated CuKα X-ray source (λ = 1.54184 Å). The data reduction process and the analytical absorption correction were performed in CrysAlis Pro [17]. For **1.2** and **5.2**, twinning was observed, and the data were processed using two grains and an orientation matrix found in CrysAlis Pro [17]. All structures were solved by direct methods and refined with the full-matrix least-squares procedure on F^2^ (SHELX-2018/1 [18]). For **1.2** and **5.2**, the flag HKLF 5 was used because of twinning. All the heavy atoms were refined with anisotropic displacement parameters. Hydrogen atoms were located at calculated positions, with the thermal displacement parameters fixed to a value 20% higher than those of the corresponding carbon atoms. For hydrogen atoms from NH groups, the positions were determined from the difference electron density synthesis. Further, **1.1** was crystallized in the triclinic P1 space group with four molecules in the asymmetric part of the unit cell. A search for higher symmetry failed to provide a model substantially worse. Hence, the proposed inversion center can be considered only as a pseudosymmetry, and the final model presented here is given in the triclinic P1 space group. All the figures were prepared in Mercury 2024.3.0 [19]. The results of the data collection and refinement are summarized in Table S2.

CCDC 2514854, 2513920, 2513921, 2513922, 2513923, and 2513924 contain the supplementary crystallographic data for **1.1**, **1.2**, **5.1**, **5.2**, **4.1**, and **4.2**, respectively. These data can be obtained free of charge from The Cambridge Crystallographic Data Centre via www.ccdc.cam.ac.uk/data_request/cif.

The intermolecular interactions were analyzed in CrystalExplorer by Hirshfeld surfaces and fingerprints [20,21,22]. The energy calculations were performed using CrystalExplorer with the B3LYP/6-31G (d,p) approach [23,24,25,26,27].

#### Spectral Characteristics of the Obtained Derivatives

Ethyl phenethylcarbamate (**1.1**)

White solid; yield: 99%; mp 32–34 °C;

^1^H NMR δ [ppm]: 1.24 (t, J = 7.04 Hz, 3 H); 2.82 (t, J = 6.90 Hz, 2 H); 3.45 (d, J = 5.87 Hz, 2 H); 4.12 (q, J = 7.04 Hz, 2 H); 4.72 (br. s., 1 H); 7.16–7.27 (m, 3 H); 7.28–7.36 (m, 2 H); ^13^C NMR δ [ppm]: 14.66; 36.19; 42.17; 60.68; 126.44; 128.59 (2xCH); 128.79 (2xCH); 138.94; 156.74. The obtained data were in good agreement with those reported in [28]. HRMS (ESI-TOF) *m*/*z*: [M + H]^+^ calculated for C_11_H_15_NO_2_ 194.1181; found 194.0771.

Ethyl (4-hydroxyphenethyl)carbamate (**2.1**)

Yellow oil; yield: 97%;

^1^H NMR δ [ppm]: 1.26 (t, J = 7.04 Hz, 3 H); 2.75 (t, J = 7.04 Hz, 2 H); 3.42 (q, J = 6.55 Hz, 2 H); 4.13 (q, J = 7.04 Hz, 2 H); 4.69 (br. s., 1 H); 5.38 (br. s., 1 H); 6.77–6.83 (m, 2 H); 7.06 (m, 2 H). Spectroscopic data were in good agreement with those reported in [29]. ^13^C NMR δ [ppm]: 14.53; 35.14; 42.43; 61.18; 115.62 (2xCH); 129.77 (2xCH); 129.96; 155.02; 157.33. HRMS (ESI-TOF) *m*/*z*: [M + H]^+^ calculated for C_11_H_15_NO_3_ 210.1130; found 210.0687.

Ethyl (2-(indolin-3-yl)ethyl)carbamate (**3.1**)

Brown oil; yield: 95%;

^1^H NMR δ [ppm]: 1.24 (t, J = 7.04 Hz, 3 H); 2.99 (t, J = 6.75 Hz, 2 H); 3.44–3.59 (m, 2 H); 4.12 (q, J = 7.04 Hz, 2 H); 4.72 (br. s., 1 H); 7.05 (d, J = 2.05 Hz, 1 H); 7.11–7.17 (m, 1 H); 7.18–7.25 m, 1 H); 7.38 (m, 1 H); 7.62 (d, J = 7.63 Hz, 1 H); 8.04 (br. s., 1 H); ^13^C NMR δ [ppm]: 14.74; 25.84; 41.41; 60.91; 111.48; 112.59; 118.73; 119.34; 122.05; 122.40; 127.40; 136.60; 157.10. Spectroscopic data were in good agreement with [30]. HRMS (ESI-TOF) *m*/*z*: [M + H]^+^ calculated for C_13_H_16_N_2_O_2_ 233.1290; found 233.0775.

Diethyl butane-1,4-diyldicarbamate (**4.1**)

Light yellow solid; yield: 98%; mp 92–93 °C;

^1^H NMR δ [ppm]: 1.15 (t, J = 7.04 Hz, 6 H); 1.40–1.54 (m, 4 H); 3.00–3.19 (m, 4 H); 4.02 (q, J = 7.04 Hz, 4 H); 5.07 (br. s., 2 H). Spectroscopic data were in good agreement with those reported in [31]. ^13^C NMR δ [ppm]: 14.56; 27.17; 40.46; 60.53; 156.80. HRMS (ESI-TOF) *m*/*z*: [M + H]^+^ calculated for C_10_H_20_N_2_O_4_ 233.1501; found 233.0793

Diethyl pentane-1,5-diyldicarbamate (**5.1**)

White solid; yield: 98%; mp 65–67 °C;

^1^H NMR δ [ppm]: 1.23 (t, J = 7.19 Hz, 6 H); 1.30–1.39 (m, 2 H); 1.48–1.55 (m, 4 H); 3.16 (q, J = 6.46 Hz, 4 H); 4.11 (q, J = 6.46 Hz, 4H); 4.71 (br. s., 2 H); ^13^C NMR δ [ppm]: 14.52; 23.64; 29.43; 40.53; 60.37; 156.84. HRMS (ESI-TOF) *m*/*z*: [M + H]^+^ calculated for C_11_H_22_N_2_O_4_ 247.1658; found 247.1102.

Ethyl (4-((ethoxycarbonyl)amino)butyl)(3-((ethoxycarbonyl)amino)propyl)carbamate (**6.1**)

Yellow oily liquid; yield: 95%;

^1^H NMR δ [ppm]: 1.19–1.31 (m, 9 H); 1.42–1.69 (m, 6 H); 3.08–3.26 (m, 8 H); 4.02–4.20 (m, 6 H); 4.71 (br. s., 1 H); 5.52 (br. s., 1 H); ^13^C NMR (101 MHz, CdCl_3_-*d*) δ [ppm]: 14.47; 20.56; 25.49; 27.06; 28.03; 37.49; 40.24; 44.03; 46.24; 53.42; 60.33; 61.09; 156.77; 156.81. HRMS (ESI-TOF) *m*/*z*: [M + H]^+^ calculated for C_16_H_31_N_3_O_6_ 362.2291; found 362.1496.

Diethyl butane-1,4-diylbis((3-((ethoxycarbonyl)amino)propyl)carbamate) (**7.1**)

Brown oil; yield: 94%;

^1^H NMR δ [ppm]: 1.18–1.32 (m, 12 H); 1.50 (br. s., 4 H); 1.69 (br. s., 4 H); 3.09–3.31 (m, 12 H); 4.09–4.16 (m, 8 H); 4.83 (br. s., 1 H); 5.52 (br. s., 1 H); ^13^C NMR δ [ppm]: 14.41; 25.04; 25.47; 27.97; 28.77; 37.45; 38.03; 43.98; 46.21; 46.46; 60.18; 60.98; 156.09; 156.66. HRMS (ESI-TOF) *m*/*z*: [M + H]^+^ calculated for C_22_H_42_N_4_O_8_ 491.3081; found 491.2216.

Ethyl (2-(1*H*-imidazol-4-yl)ethyl)carbamate (**8.1**)

Light yellow oil, yield: 70%;

^1^H NMR δ [ppm]: 1.44 (t, J = 7.19 Hz, 3 H); 3.50 (q, J = 6.16 Hz, 2 H); 4.10 (q, J = 6.85 Hz, 2 H); 4.47 (q, J = 7.04 Hz, 2 H); 5.20 (br. s., 1 H); 7.22 (s, 1 H); 8.13 (s, 1 H); ^13^C NMR δ [ppm]: 14.64; 27.98; 40.05; 64.58; 113.88; 136.75; 141.05; 156.67. HRMS (ESI-TOF) *m*/*z*: [M + H]^+^ calculated for C_8_H_13_N_3_O_2_ 184.1086; found 184.0705

2,2,2-Trifluoroethyl phenethylcarbamate (1.2)

White solid; yield: 99%; mp 34–36 °C;

^1^H NMR δ [ppm]: 2.84 (t, J = 7.04 Hz, 2 H); 3.49 (q, J = 8.51 Hz 2 H); 4.45 (q, J = 8.51 Hz, 2 H); 4.92 (br. s., 1 H); 7.19 (d, J = 7.04 Hz, 2 H); 7.22–7.28 (m, 1 H); 7.29–7.35 (m, 2 H); ^13^C NMR δ [ppm]: 35.84; 42.43; 60.82 (q, J = 38.92 Hz, OCH_2_); 123.10 (q, J = 277.82 Hz, CF_3_); 126.68; 128.72; 128.73; 138.29; 154.33; ^19^F NMR δ [ppm]: −75.30. Spectroscopic data were in good agreement with [32]. HRMS (ESI-TOF) *m*/*z*: [M + H]^+^ calculated for C_11_H_12_F_3_NO_2_ 248.0898; found 248.0358.

2,2,2-Trifluoroethyl (4-hydroxyphenethyl)carbamate (**2.2**)

Yellow oil; yield: 97%;

^1^H NMR δ [ppm]: 2.76 (t, J = 6.90 Hz, 2 H); 3.39–3.48 (m, 2 H); 4.45 (q, J = 8.51 Hz, 2 H); 4.91 (br. s., 1 H); 5.02 (br. s., 1 H); 6.74–6.82 (m, 2 H); 7.04–7.06 (m, 2 H); ^13^C NMR δ [ppm]: 34.94; 42.63; 60.87 (q, J = 35.83 Hz, OCH_2_); 115.58 (2xCH); 123.09 (q, J = 276.66 Hz, CF_3_); 129.89 (2xCH); 130.31; 147.46; 154.42; ^19^F NMR δ [ppm]: −75.30. HRMS (ESI-TOF) *m*/*z*: [M + H]^+^ calculated for C_11_H_12_F_3_NO_3_ 264.0848; found 264,0284.

2,2,2-Trifluoroethyl (2-(indolin-3-yl)ethylcarbamate (**3.2**)

White solid; yield: 98%; mp 51–53 °C;

^1^H NMR δ [ppm]: 3.03 (t, J = 6.80 Hz, 2 H); 3.58 (q, J = 6.67 Hz, 2 H); 4.48 (q, J = 8.54 Hz, 2 H); 4.99 (br. s., 1 H); 7.07 (d, J = 2.13 Hz, 1 H); 7.16–7.18 (m, 1 H); 7.24–7.26 (m, 1 H); 7.38–7.44 (m, 1 H); 7.62 (d, J = 7.74 Hz, 1 H); 8.07 (br. s., 1 H); ^13^C NMR δ [ppm]: 25.54; 41.52; 60.81 (q, J = 36.14 Hz, OCH_2_); 111.27; 112.58; 118.67; 119.64; 122.10; 122.36; 121.10 (q, J = 278,20 Hz, CF_3_); 127.21; 136.44; 154.41; ^19^F NMR δ [ppm]: −75.27. HRMS (ESI-TOF) *m*/*z*: [M + H]^+^ calculated for C_13_H_13_F_3_N_2_O_2_ 287.1007; found 287.0523.

Bis(2,2,2-trifluoroethyl) butane-1,4-diyldicarbamate (**4.2**)

Light yellow solid; yield: 99%; mp 117–118 °C;

^1^H NMR δ [ppm]: 1.45–1.75 (m, 4 H); 3.24 (d, J = 6.16 Hz, 4 H); 4.45 (q, J = 8.51 Hz, 4 H); 4.99 (br. s., 2 H); ^13^C NMR δ [ppm]: 26.93; 40.81; 60.89 (q, J = 36.61 Hz, OCH_2_); 123.10 (q, J = 279.00 Hz, CF_3_); 154.52; ^19^F NMR δ [ppm]: −75.32. HRMS (ESI-TOF) *m*/*z*: [M + H]^+^ calculated for C_10_H_14_F_6_N_2_O_4_ 341.0936; found 341.0200.

Bis(2,2,2-trifluoroethyl)pentane-1,5-diyldicarbamate (**5.2**)

White solid; yield: 98%, mp 97–99 °C;

^1^H NMR δ [ppm]: 1.29–1.43 (m, 2 H); 1.49–1.62 (m, 4 H); 3.22 (q, J = 6.75 Hz, 4 H); 4.45 (q, J = 8.51 Hz, 4 H); 4.97 (br. s., 2 H); ^13^C NMR δ [ppm]: 23.39; 29.20; 40.93; 60.86 (q, J = 36.99 Hz, OCH_2_); 123.13 (q, J = 277.82 Hz, CF_3_); 154.56; ^19^F NMR δ [ppm]: −75.36. HRMS (ESI-TOF) *m*/*z*: [M + H]^+^ calculated for C_11_H_16_F_6_N_2_O_4_ 355.1093; found 355.0322.

2,2,2-Trifluoroethyl (4-(((2,2,2-trifluoroethoxy)carbonyl)amino)butyl)(3-(((2,2,2-trifluoroethoxy)carbonyl)amino)propyl)carbamate (**6.2**)

Yellow oil; yield: 96%;

^1^H NMR δ [ppm]: 1.40–1.67 (m, 6 H); 3.16–3.40 (m, 8 H); 4.36–4.58 (m, 6 H); 5.13 (br. s., 1 H); 5.69 (br. s., 1 H); ^13^C NMR δ [ppm]: 25.45; 27.00; 27.86; 37.92; 40.66; 44.75; 46.76; 61.14 (q, J = 37.80 Hz, OCH_2_); 123.11 (q, J = 280.10 Hz, CF_3_); 154.16; 154.58; 154.97; ^19^F NMR δ [ppm]: −75.26. HRMS (ESI-TOF) *m*/*z*: [M + H]^+^ calculated for C_16_H_22_F_9_N_3_O_6_ 524.1443; found 524.0312.

Bis(2,2,2-trifluoroethyl) butane-1,4-diylbis((3-(((2,2,2-trifluoroethoxy)carbonyl)amino)propyl)carbamate) (**7.2**)

Light brown oil; yield: 97%;

^1^H NMR δ [ppm]: 1.55 (br. s., 4 H); 1.69–1.86 (m, 4 H); 3.15–3.36 (m, 12 H); 4.40–4.57 (m, 8 H); 5.68 (br. s., 2 H); ^13^C NMR δ [ppm]: 25.38; 25.63; 27.78; 28.82; 37.83; 38.56; 44.42; 44.65; 46.76; 47.31; 60.80 (q, J = 36.80 Hz, OCH_2_); 61.51 (q, J = 36.80 Hz, OCH_2_); 123.15 (q, J = 280.40 Hz, CF_3_); 154.57; 154.96; ^19^F NMR δ [ppm]: −75.29. HRMS (ESI-TOF) *m*/*z*: [M + H]^+^ calculated for C_22_H_30_F_12_N_4_O_8_ 707.1950; found 707.0415.

NMR and HR-MS spectra are presented in the Supplementary Materials.

### 2.4. GC Analysis

The volatility of the obtained derivatives, except for **8.1** and **8.2**, was analyzed using a gas chromatograph, model 5890 Series II, Hewlett Packard, coupled with a flame ionization detector (FID) and a ZB-WAX column (15 m × 0.54 μm, GC-FID). Two separate mixtures, each containing seven derivatives with a concentration of 1.67 g/L in methanol, were prepared. The conditions for ECF and TFECF derivatives analysis are presented as follows: carrier gas (helium) flow 0.35 [mL/min]; injector temperature 220 °C; detector temperature 230 °C; temperature program 200 °C–12 min, 5 °C/min–240 °C–60 min.

The GC-MS analysis was performed using the gas chromatograph Shimadzu GC-MS-TQ8040 with the ZB-WAX column (30 m × 0.25 μm) coupled with the Shimadzu MASS spectrometer GC-2010 Plus (electron ionization). The injection was performed at a pressure of 130 kPa and 220 °C; detector temperature 230 °C; temperature program 200 °C–12 min, 5 °C/min–240 °C–60 min, and carrier gas flow was 10 [mL/min]. The data were collected using LabSolutions GC-MS Solution Version 4.45.

### 2.5. Sample Preparation

The authors selected three fermented vegetable juices commonly available in an organic food store for analysis: sample 1 (fermented cucumber juice, S1), sample 2 (fermented cabbage juice, S2), and sample 3 (fermented tomato juice, S3). All juices (five bottles of each) originated with a single manufacturer operating in central Poland.

Juice samples were mixed on a magnetic stirrer for 15 min, filtered through a soft filter paper, and remixed. Five laboratory samples (10 mL) of each juice were collected for further analysis, transferred to round-bottom flasks, and adjusted to pH 10 with solid NaOH (pH was monitored with a pH meter). Subsequently, 5 mL of EtOAc and 0.2 mL of TFECF or 5 mL of Et_2_O and 0.20 mL of ECF, respectively, were added to the flasks. The reaction mixture was maintained at 5 to10 °C for 15 min and then stirred for an additional 2 h at room temperature. Next, the resulting mixtures were then extracted with EtOAc (3 × 10 mL), dried over anhydrous MgSO_4_, and evaporated under reduced pressure. Finally, the resulting oily residue was dissolved in methanol (for GC-MS) in a 10 mL volumetric flask.

### 2.6. Statistical Evaluation of the GC-MS Procedure

The studied procedure for phenylethylamine determination was evaluated using selected validation parameters: linearity (coefficient of determination, R^2^), detection limit (DL), quantification limit (QL), precision (coefficient of variation, CV), and accuracy (recovery rate). Linear ranges of the obtained curves for selected derivatives (7 different concentrations) were recorded and converted into pure biogenic amine concentrations. Intra-day precision and recovery were determined based on three replicates of selected derivative samples at three concentrations (corresponding to the minimum, intermediate, and maximum values of the standard calibration curves) for one day. The detection sensitivity was evaluated as response factors (RFs), followed by the calculated relative response factors (RRFs). The matrix effect (ME) for the proposed phenylethylamine determination procedures was calculated by adding this amine to selected juice samples (fermented cabbage juice, S2) at five different concentrations.

For each tested juice, five samples were used for derivatization procedures, and the results were calculated as the mean ± standard deviation.

One-way analysis of variance (ANOVA), followed by Tukey’s post hoc test, was performed to analyze the significant differences between the data (*p* < 0.05; Statistica 8 software, StatSoft, Tulsa, OK, USA). Moreover, to assess the effect of the derivatization reagent used (ECF or TFECF) on GC–MS results, a two-factor analysis of variance was applied.

## 3. Results and Discussion

The choice of the appropriate derivatization agent depends on the reactivity of the compound—reagents with extended R groups are less reactive but can form stable derivatives. The most commonly described reactions employ apolar reagents, which necessitate the use of organic solvents. In contrast, chloroformates enable the rapid derivatization of amines at room temperature, converting them into carbamates in buffered aqueous media [33,34]. Acylation reduces the polarity of hydroxyl, amino, and thiol groups. Compared with silylation, acylation reagents are suitable for highly polar, multifunctional compounds, such as BAs. Chloroformates containing simple alkyl groups, such as ethyl, are well-established as effective reagents for derivatizing amino groups in gas chromatography and can be regarded as potential general-purpose reagents in analytical chemistry [33]. Their principal advantages include rapid reaction kinetics, no requirement for rigorous water removal, low reagent cost, and simplified sample preparation workflows. Although reagents from this group of compounds are hazardous and scarce [3], their advantages make them a promising alternative for the determination of BAs using GC.

In this study, we evaluated 2,2,2-trifluoroethyl chloroformate (TFECF) and ethyl chloroformate (ECF)—two structurally related chloroformate reagents that differ markedly in volatility, hazard profile, and chemical reactivity. We expected that incorporating fluorine atoms into the TFECF molecule would enhance both the stability and volatility of the resulting biogenic amine derivatives.

**Figure 2:**
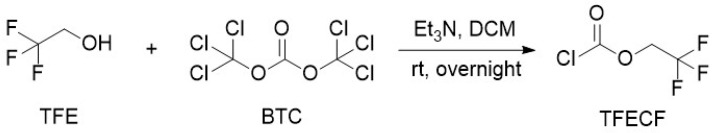
The synthesis of TFECF.

The first stage of the work involved safely synthesizing TFECF using 2,2,2-trifluoroethanol and bis(trichloromethyl)carbonate (BTC), also known as triphosgene, a crystalline, safe replacement for gaseous phosgene, as starting materials (Figure 2) [35,36].

**Figure 3:**
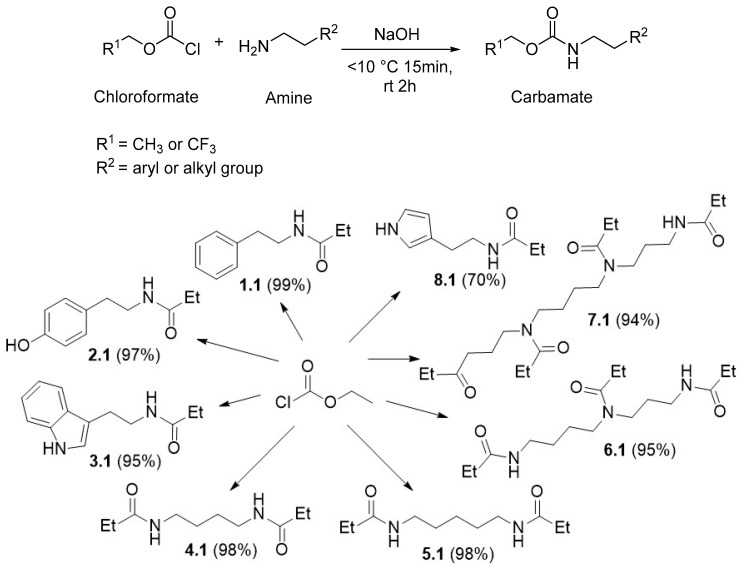

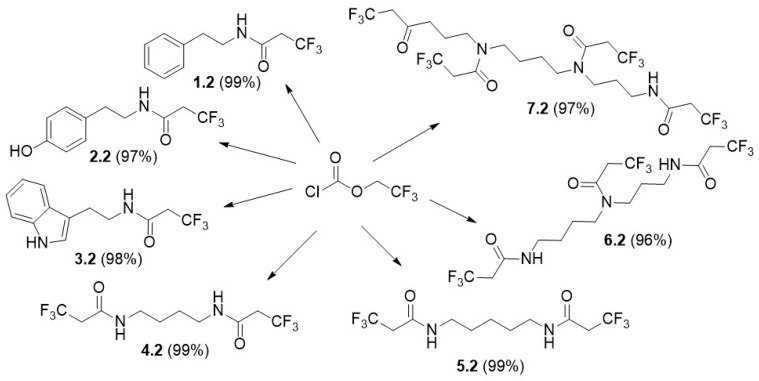
Reaction scheme of BAs with chloroformates, with the structural formulas of the resulting products.

The next stage of the study involved optimizing the reaction conditions of carbamate formation. Several methods for obtaining such derivatives have been described in the literature [37,38,39]. In our study, we proposed conducting the reaction in a solvent system of EtOAc/H_2_O (1:2) for TFECF and Et_2_O/H_2_O (1:2) for ECF (for details on the selection of the solvent system for the synthesis of derivatives, please refer to Supplementary Materials: Table S5). Since hydrochloric acid is generated during the reaction, sodium hydroxide was added in a 1.1 molar equivalent relative to the amino group. Under the proposed conditions, eight derivatives for ECF (**1.1**–**8.1**) and seven for TFECF (**1.2**–**7.2**) were successfully obtained, exhibiting excellent yields (Figure 3). For the **8** TFECF case, we could not confirm the structure of the obtained derivative. The NMR spectra revealed the presence of numerous byproducts and impurities. Consequently, in the applied section of the study, we discontinued further investigation of the histamine derivatives.

The structural characterization of products (^1^H, ^13^C, and ^19^F NMR and MS spectra) confirms their structures and high purity. Additionally, for the compounds obtained as solids that yielded suitable crystals, crystallographic characterization was performed.

**Figure 4:**
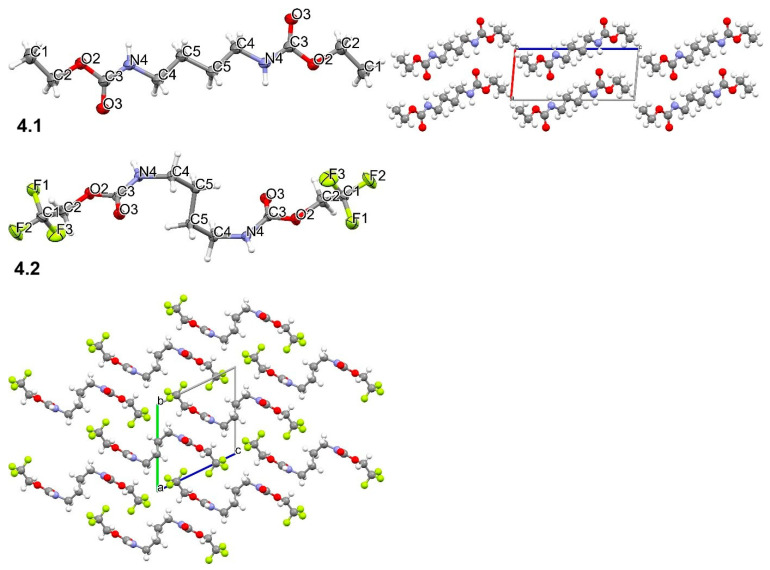
The structure of **4.1** and **4.2** with ellipsoids at the level of 50% probability and the numbering scheme (**left column**) and packing motifs (**right column**).

Putrescin carbamate **4.1** crystallizes in the triclinic P-1 space group with all atoms found in the general positions and a half of the molecule in the asymmetric part of the unit cell (Figure 4). The molecule occurs in an extended conformation, with all torsion angles close to 0° or 180° (Table S3). In the packing, columns running along *an* axis are observed with molecules connected by N-H∙∙∙O hydrogen bonds. In contrast, interactions between adjacent columns are assured by dispersion forces (Figure 4). It is shown by projection of intermolecular interactions onto Hirshfeld surfaces—the weak H∙∙∙H contacts prevail, but H∙∙∙O distances are also significant, with some short distances (red spots on Hirshfeld surfaces and spikes on the fingerprints) corresponding to hydrogen bonds (Figure S1). In the energy calculation, it is evident that the dominant electrostatic interactions between molecules in the column are mediated by a hydrogen bond (Figure S2). In other directions, dispersion interactions make a significant, and even prevailing, contribution.

Carbamate **4.2** crystallizes in the triclinic P-1 space group with all atoms found in the general positions and a half of the molecule in the asymmetric part of the unit cell (Figure 4). The molecule is strongly twisted with torsion angles being −110.21 (11) and 65.38 (14) for C3-N4-C4-C5 and N4-C4-C5-C5[-x+1,-y+1,-z], respectively (Table S4). It can be described as a chair configuration. Hence, the CF_3_ group introduced significant structural changes and also affected the packing motif. The main feature, namely N-H∙∙∙O hydrogen bonds formed between molecules that form a column along the *a* axis, is maintained. The red spots are positioned at Hirshfeld surfaces and spikes on fingerprints, indicating N-H∙∙∙O hydrogen bonds are clearly visible (Figure S3). Hence, those parts of adjacent molecules are similarly oriented in **4.1** and **4.2**. However, the shape of the molecules imposes different interactions. CF_3_ groups penetrate the surface groove, and the number of H∙∙∙H contacts is substantially lowered, being replaced by H∙∙∙F and F∙∙∙F contacts. In the former case, they are rather long and do not involve hydrogen bonds. In the energy pattern, the main interactions arise from molecules in the column, which are connected by strong electrostatic forces from hydrogen bonding. Interactions between molecules from adjacent layers are significantly weaker and are mostly dispersive (Figure S4).

Summarizing, all crystalline compounds occur in rather extended conformation, and the most twisted conformation is observed for **4.2**. In the packing, columns with molecules connected by N-H⋯O hydrogen bonds are the common supramolecular motif, whereas adjacent columns interact only by weak van der Waals forces. For TFECF derivatives, fluorine atoms are not involved in any hydrogen bonds, and only weak H…F interactions are detected. For **1.2**, three independent molecules were found in the asymmetric part of the unit cell, and they form one column with the molecule sequence …ABCABC… running along the *b* axis. In **1.1**, there are four molecules in the asymmetric part of the unit cell grouped in two columns with …ABAB… and …CDCD… sequences (Figure S5).

Analysis of the obtained carbamates using gas chromatography with a flame ionization detector (FID) enabled a comparison of the volatility and separation capabilities of the tested compounds. For six BA derivatives from ECF and TFECF (**1.1**–**6.1** and **1.2**–**6.2**, respectively), FID signals were recorded, and the retention times of the obtained compounds are presented in Table 1. Additionally, chromatograms of the mixtures of the obtained derivatives are presented in the Supplementary Materials (Figures S6 and S7). In the case of **7.1** and **7.2** (spermine derivatives), despite repeatedly performed experiments, it was not possible to register a signal. It was most likely due to the high molecular mass of the obtained derivative, resulting from the presence of four functional groups. Interestingly, the presence of four trifluoromethyl groups in the structure of compound **7.2** adversely affected its volatility, presumably because of the increased contact surface area and the resulting enhancement of van der Waals interactions.

The obtained results confirm the satisfactory volatility of the new derivatives and, consequently, the feasibility of their application in the quantitative analysis of BAs in real samples.

### Application Aspect of the Obtained Derivatives

As described above, the most commonly occurring BAs in foods include tyramine, histamine, phenylethylamine, tryptamine, cadaverine, putrescine, spermidine, and spermine [4,7]. In this paper, we focused on the determination of phenylethylamine (**1**) in selected fermented vegetable juices. Fermented foods are recognized as essential dietary components and are characterized by high amino acid availability and microbial activity, which can promote the formation of biogenic amines [7]. Moreover, the choice of phenylethylamine was supported by the fact that analytical studies on fermented foods typically focus on histamine, tyramine, putrescine, and cadaverine, which are generally present at higher concentrations. In contrast, phenylethylamine is normally present at trace levels [40,41]. Due to the trace amounts of the tested amine in the food, GC-MS was proposed for quantitative analysis.

**Figure 5:**
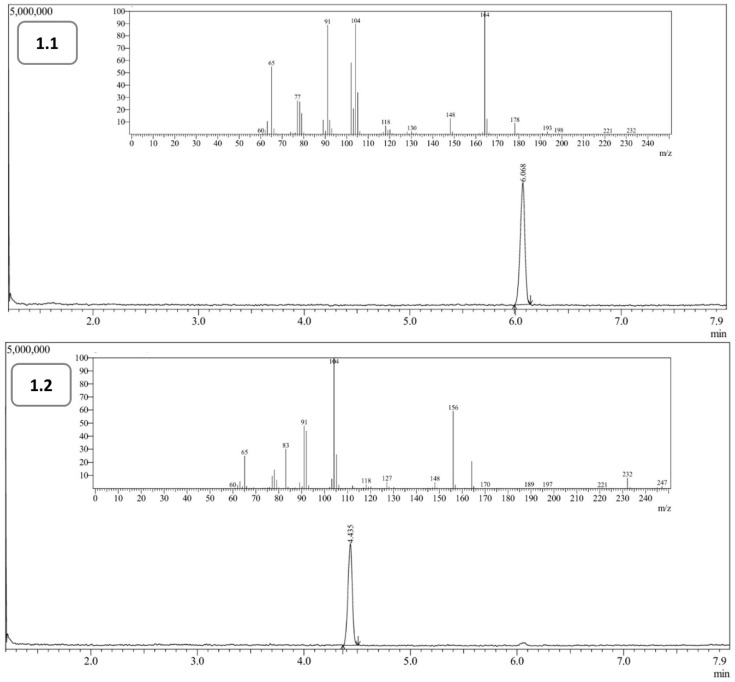
The chromatograms of **1.1** and **1.2** with the MS spectra.

The regression parameters of the calibration curves for the **1.1** and **1.2** compounds are listed in Table 2. Representative chromatograms of the obtained derivatives, together with the corresponding MS spectra, are shown in Figure 5.

The reproducibility of the retention time analysis, expressed as the intra-day coefficient of variation, was 0.068% and 0.073% for **1.1** and **1.2**, respectively. The determined results regarding the linearity of the method, as well as limits of detection and quantification, suggest suitable linearity of the tested concentration range for the discussed derivatives. Moreover, the precision and accuracy of the proposed procedure were satisfactory and similar, regardless of the reagent used for the synthesis.

The final statistical parameter evaluated was the matrix effect on the analytical signals obtained. Example chromatograms with MS spectra illustrating the matrix effect are presented in the Supplementary Materials (Figures S8 and S9). The calculated ME values were low, amounting to approximately +5% for ECF and −9% for TFECF. Although the number of analyzed samples was limited, the low matrix effects (<20%) indicate negligible matrix-related signal interference.

Based on the satisfactory validation parameters obtained for the proposed method, a detailed evaluation of analytical sensitivity was subsequently carried out to compare ECF and TFECF derivatives. Analytical sensitivity of the GC–MS method was assessed by comparing detector responses for both derivatives under identical experimental conditions (Table S6, Supplementary Materials). The results demonstrated that TFECF derivatives yielded higher detector responses than the corresponding ECF products. The calculated relative response factors were concentration-independent and showed good repeatability, with a mean value of 1.31 ± 0.06 (n = 7). This approximately 30% increase in detector response clearly indicates that TFECF enhances the analytical sensitivity of the proposed GC–MS procedure.

To further evaluate the influence of the derivatization reagent on the analytical results, a two-factor ANOVA was applied (Table S7, Supplementary Materials). A statistically significant effect of the derivatization reagent on peak area was observed (*p* < 0.001), while analyte concentration also significantly influenced detector response (*p* < 0.001). Importantly, no statistically significant interaction between the derivatization reagent and concentration was found (*p* > 0.05), indicating that the advantage of TFECF over ECF was maintained across the entire concentration range investigated.

Finally, analytical sensitivity was additionally evaluated using the signal-to-noise ratio (SNR). The mean log(SNR) values for ECF and TFECF derivatives were 57.36 ± 3.45 and 62.00 ± 3.01, respectively, further confirming the superior sensitivity of TFECF derivatization.

**Figure 6:**
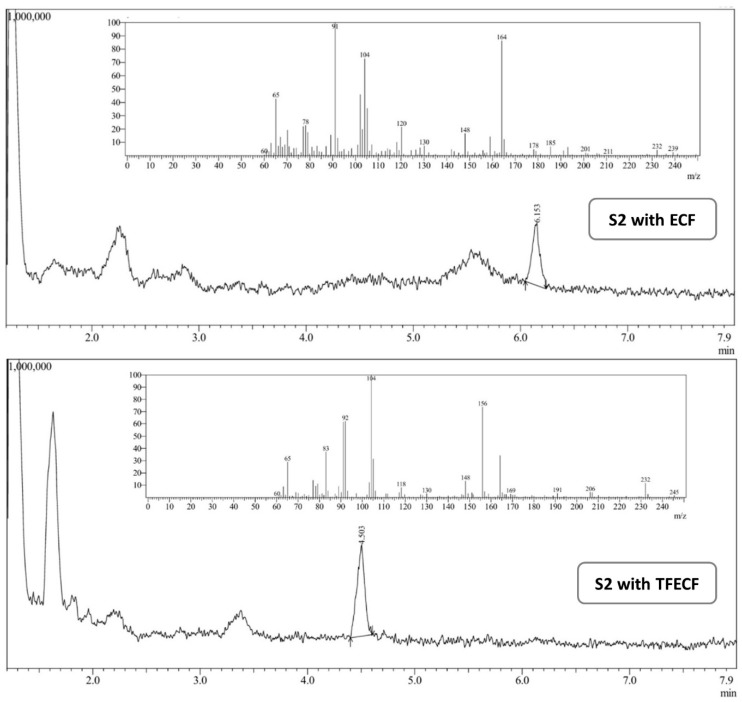
The chromatograms of sample S2 with the MS spectra.

The developed procedures were used to analyze phenylethylamine content in selected fermented vegetable juices. Example chromatograms with MS spectra are shown in Figure 6, and the obtained results are summarized in Table 3. The statistical evaluation is presented in the Supplementary Materials (Table S8).

Due to the very low concentration of the tested compound in sample S3, the standard-addition method was used for quantitative analysis rather than an external calibration curve.

The results showed apparent differences in phenylethylamine concentration across the tested juices. Moreover, these differences were observed regardless of the derivatization reagent used. It should be noted that limited literature on phenylethylamine in fermented vegetable juices is available. In our previous study [42], we analyzed the content of selected biogenic amines, including phenylethylamine, in various fermented vegetable juices. This biogenic compound was quantified in only four samples, including cucumber, cauliflower, red cabbage, and celery, as well as beetroot juice, at levels ranging from 0.072 mg/L to 0.872 mg/L, respectively. The values obtained in the present study are considerably higher; however, they are difficult to compare because different raw materials and technological processes were used.

Statistical analysis of the observed differences between the obtained results was performed using one-way analysis of variance (ANOVA), followed by Tukey’s post hoc test (*p* < 0.05). The developed test revealed a statistically significant difference between the mean values obtained for samples S1 and S2. Additionally, the effects of derivatization reagent and sample type on the analytical response were evaluated using fixed-effects two-way ANOVA (Table S8, Supplementary Materials).

The two-way ANOVA revealed a statistically significant effect of the derivatization reagent (*p* < 0.001) and a substantial impact of sample type (*p* < 0.001). In addition, a statistically significant interaction between the derivatization reagent and the sample was observed (*p* < 0.001), indicating that the effect of the derivatization reagent varied across sample matrices. Moreover, post hoc analysis using Tukey’s test showed statistically significant differences between applied reagents within individual samples (*p* = 0.0001824), although the magnitude of these differences was sample-dependent. Although the derivatization reagent had a statistically significant effect on the analytical response, the presence of a substantial reagent × sample interaction and the limited number of analyzed samples suggest that the observed differences should be interpreted with caution. Nevertheless, the results indicate a consistent trend toward higher responses to TFECF than to ECF across the investigated samples.

In summary, despite the limited number of analyzed samples, the observed trends indicate the suitability of the proposed procedure based on the derivatization of phenylethylamine with TFECF and its GC-MS determination in complex food matrices. Our further research on the application of the developed method will focus on checking its applicability to other biogenic amines and a larger number of tested food samples.

## 4. Conclusions

This work introduces a robust derivatization strategy for the selected biogenic amines based on the formation of fluorinated carbamates with 2,2,2-trifluoroethyl chloroformate, enabling highly efficient GC analysis and demonstrating clear advantages over conventional ethyl chloroformate. The newly obtained derivatives exhibited satisfactory volatility, good chromatographic separation, and analytical precision in real-sample applications. The latter was suggested by the determination of phenylethylamine in fermented vegetable juices.

However, some issues still need to be addressed. The derivatization of spermine, a tetrafunctional polyamine, yielded derivatives with insufficient volatility for GC-FID detection. Looking forward, improving derivative stability and enabling the efficient derivatization of higher-order polyamines should be prioritized.

## Figures and Tables

**Table 1:** The retention times (RT) of the obtained derivatives.

BAs Derivatives	RT [min]	BAs Derivatives	RT [min]
**1.1**	2.17	**2.1**	1.56
**2.1**	11.53	**2.2.**	6.87
**3.1**	14.57	**3.2**	8.61
**4.1**	23.44	**4.2**	20.28
**5.1**	39.22	**5.2**	24.54
**6.1**	45.73	**6.2**	29.48

**Table 2:** Validation parameters of calibration curves obtained by GC-MS techniques.

	T_[min]_ ±SD	Range [mg/L]	R^2^	DL [mg/L]	QL [mg/L]	Rec ±SD [%]	Intra-Day Precision [CV, %]*	ME
**1.1**	6.06 ± 0.004	1.0–10.0	0.9964	0.51	1.55	96 ± 9.33 *	4.62 *	5.22
**1.2**	4.43 ± 0.003		0.9964	0.53	1.61	97 ± 11.98 *	3.97 *	−9.05

Where T—migration time; R^2^—coefficient of determination; DL (Detection Limit) [mg·L^−1^] = (3 × s_y/x_) × b^−1^; QL (Quantification Limit) [mg·L^−1^] = (10 × s_y/x_) × b^1−^; Rec—recovery was calculated as mean value of (experimental concentration/found concentration) 100%; SD—standard deviation, CV—coefficient of variation. * Average value for three concentrations analyzed in triplicate during one day. ME—matrix effect calculated as: (*a*_slope of the curve in the matrix_/*a*_slope of the curve in the solvent_ − 1)·100%.

**Table 3:** Results of the analysis of derivatives **1.1** and **1.2** are expressed as the free phenylethylamine in the food samples.

Sample	TFECF		ECF		One-Way Anova
	X ± SD [mg/L]	CV [%]	X ± SD [mg/L]	CV [%]	*p*
S1	1.2738 ± 0.0364	2.85	1.4840 ± 0.1008	6.79	0.002329
S2	6.7815 ± 0.1028	1.52	5.7414 ± 0.3777	6.58	0.0003456
S3	0.5096 ± 0.0230	4.52	0.5318 ± 0.0527	8.03	0.337

## Data Availability

The original contributions presented in this study are included in the article/Supplementary Material. Further inquiries can be directed to the corresponding authors.

## Supplementary Materials

The following supporting information can be downloaded at https://www.mdpi.com/article/10.3390/ma19030575/s1, Table S1: Eluents used for the purification of BA’s derivatives by column chromatography; Table S2: The results of the data collections and refinement for **1.1**, **1.2**, **5.1**, **5.2**, **4.1**, and **4.2**; Table S3: Torsion angles in **4.1**; Figure S1: Hirshfeld surfaces (left) and fingerprints (right) of selected intermolecular interactions found in the crystal network **4.1**: (a) for H⋯O (27.1%), (b) for H⋯H (64.6%). In brackets the surface area is given as a percentage of the total surface area; Figure S2: Interactions energy (total in blue, electrostatic in red and dispersion in green) for **4.1**; Table S4: Torsion angles in **4.2**; Figure S3: Hirshfeld surfaces (left) and fingerprints (right) of selected intermolecular interactions found in the crystal network **4.2**: (a) for H⋯O (18.4%), (b) for H⋯H (19.4%), (c) for H⋯F (45.9%), (d) for F⋯F (9.0%). In brackets the surface area is given as a percentage of the total surface area; Figure S4: Interactions energy (total in blue, electrostatic in red and dispersion in green) for **4.2**; Figure S5: The structure of **1.1**, **1.2**, **5.1**, and **5.2** with ellipsoids at the level of 50% probability and the numbering scheme (left column) and packing motifs (right column); Table S5: Selection of solvent composition for the reaction of phenylethylamine with ECF; Figure S6: Chromatogram of the mixture of the obtained ECF derivatives of the tested BAs, where: **1.1**—Phen-ECF; **2.1**—Tyr-ECF; **3.1**—Trp-ECF; **4.1**—Put-ECF; **5.1**—Cad-ECF; **6.1**—Spd-ECF; Figure S7: Chromatogram of the mixture of the obtained TECF derivatives of the tested BAs, where: **1.2**—Phen-TFECF; **2.1**—Tyr-TFECF; **3.1**—Trp-TFECF; **4.1**—Put-TFFCF; **5.1**—Cad-TFECF; **6.1**—Spd-TFECF; Figure S8:Chromatogram (GC-MS) of the matrix with standard addition (**1.1**) and pure standard (**1.1**); Figure S9: Chromatogram (GC-MS) of the matrix with standard addition (**1.2**) and pure standard (**1.2**); Table S6: The mean RF and RFF value for the determination of phenylethylamine derivatives (n = 7); Table S7: Results of two-factor ANOVA for the evaluation of the effect of derivatization reagent on GC–MS analytical results; Table S8: Results of fixed-effects two-way ANOVA evaluating the effects of derivatisation reagent and sample type on the analytical response; Figure S10: HRMS spectra of the obtained derivatives.
