# Supplementary material for: Synthesis of New Volatile Derivatives of Biogenic Amines, Carbamates for Analytical Applications
Source: Materials (Basel). 2026 Feb 2;19(3):575. doi: 10.3390/ma19030575 (PMC12898731; doi:10.3390/ma19030575)

Synthesis of new volatile derivatives of biogenic amines, carbamates for analytical applications

Kamil Brzuzy[1,2], Aneta Jastrzębska[1*], Anna Kmieciak[1], Jacek Ścianowski[1], Tadeusz Muzioł[1], Damian Gorczyca[3,4], Marek P. Krzemiński[1*]

[1]Faculty of Chemistry, Nicolaus Copernicus University in Toruń, 7 Gagarin Str., 87-100 Toruń, Poland

[2]Fresh Inset, Wileńska 4/A017 Str., 87-100 Toruń, Poland

[3]Faculty of Medicine, Vizja University, Okopowa 59 Str., 01-043, Warsaw, Poland

[4]LymeLab Pharma Sp. z o.o. Sp. k., Kochanowskiego 49A Str., 01-864 Warsaw, Poland

* Corresponding authors: Aneta Jastrzębska, Marek P. Krzemiński

Supplementary Materials

Table S1. Eluents used for the purification of BA's derivatives by column chromatography

	Eluent	
BA's	ECF derivative	TFECF derivative
**1**	DCM + 1% MeOH	DCM + 3% MeOH
**2**	Heksan/EtOAc (8020) + 1% MeOH	DCM + 3% MeOH
**3**	Heksan/EtOAc (80/20) + 1% MeOH	DCM + 3% MeOH
**4**	DCM + 10% MeOH	DCM + 3% MeOH
**5**	DCM + 10% MeOH	DCM + 3% MeOH
**6**	DCM + 5% MeOH	DCM + 2% MeOH
**7**	DCM + 5% MeOH	DCM + 2% MeOH
**8**	Heksan/EtOAc (70/30) + 2% MeOH	DCM + 2% MeOH

Where: DCM - dichloromethane, EtOAc - ethyl acetate, MeOH - methanol

Table S2. The results of the data collections and refinement for **1.1**, **1.2**, **5.1**, **5.2**, **4.1**, and **4.2**.

Identification code	**1.1**	**1.2**	**5.1**	**5.2**	**4.1**	**4.2**
Empirical formula	$C_{11} H_{15} N O_2$	$C_{11} H_{12} F_3 N O_2$	$C_{11} H_{22} N_2 O_4$	$C_{11} H_{16} F_6 N_2 O_4$	$C_{10} H_{20} N_2 O_4$	C10 H14 F6 N2 O4
Formula weight	193.24	247.22	246.30	354.26	232.28	340.23
Temperature [K]	100(2)	100(2)	100(2)	100(2)	100(2)	100(2)
Wavelength [Å]	1.54184	1.54184	1.54184	1.54184	1.54184	1.54184
Crystal system, space group	Triclinic, P1	Monoclinic, C2/c	Monoclinic, $P2_1/c$	Monoclinic, $P2_1/m$	Triclinic, P-1	Triclinic, P-1
Unit cell dimensions [Å] and [°]	a = 7.6967(4)<br>α = 73.068(4).<br>b = 9.9644(5)<br>β = 81.148(4)<br>c = 14.8416(7)<br>γ = 89.771(4)°	a = 16.4397(5)<br>α = 90<br>b = 14.7918(3)<br>β = 102.051(3)<br>c = 28.5732(8)<br>γ = 90	a = 19.1875(2)<br>α = 90<br>b = 5.01690(5)<br>β = 95.7866(10)<br>c = 14.26180(16)<br>γ = 90	a = 4.84804(11)<br>α = 90<br>b = 19.3597(5)<br>β = 98.495(3)<br>c = 8.2032(2)<br>γ = 90	a = 4.9977(3)<br>α = 100.174(5)<br>b = 5.1707(3)<br>β = 94.672(5)<br>c = 12.2482(6)<br>γ = 91.351(5)	a = 5.03660(10)<br>α = 64.189(3)<br>b = 8.8249(3)<br>β = 84.991(2)<br>c = 8.8343(3)<br>γ = 87.354(2)
Volume [Å^3]	1074.93(9)	6795.1(3)	1365.87(3)	761.48(4)	310.27(3)	352.12(2)
Z, calculated density [Mg/m^3]	4, 1.194	24, 1.450	4, 1.198	2, 1.545	1, 1.243	1, 1.604
Absorption coefficient [mm^{-1}]	0.662	1.156	0.751	1.458	0.797	1.550
F(000)	416	3072	536	364	126	174
Crystal size [mm^3]	0.14 x 0.05 x 0.03	0.080 x 0.023 x 0.019	0.19 x 0.09 x 0.07	0.27 x 0.02 x 0.017	0.28 x 0.08 x 0.04	0.19 x 0.04 x 0.02
Theta range for data collection [°]	3.153 to 86.634.	3.163 to 74.501.	2.314 to 74.464.	4.568 to 74.482.	3.680 to 74.461	5.569 to 74.369

Index ranges	-9<=h<=9, -12<=k<=12, -18<=l<=18	-20<=h<=20, -18<=k<=18, -35<=l<=35	-23<=h<=23, -6<=k<=6, -17<=l<=17	-5<=h<=5, -24<=k<=24, -10<=l<=10	-4<=h<=6, -6<=k<=6, -15<=l<=15	-4<=h<=6, -9<=k<=11, -11<=l<=10
Reflections collected/unique	27078, 7887 [R(int) = 0.0628]	9914	20273, 2786 [R(int) = 0.0265]	2383	3280, 1251 [R(int) = 0.0315]	3823, 1429 [R(int) = 0.0189]
Completeness [%] to theta [°]	67.684 99.6 %	67.684 99.7 %	67.684 100.0 %	67.684 99.6 %	67.684 99.6 %	67.684 100.0 %
Absorption correction	Gaussian	Gaussian	Gaussian	Gaussian	Gaussian	Gaussian
Max. and min. transmission	1.000 and 0.852	0.980 and 0.937	1.000 and 0.748	0.979 and 0.826	1.000 and 0.704	1.000 and 0.778
Refinement method	Full-matrix least-squares on F^2	Full-matrix least-squares on F^2	Full-matrix least-squares on $F^2$2	Full-matrix least-squares on F^2	Full-matrix least-squares on F^2	Full-matrix least-squares on F^2
Data / restraints / parameters	7887 / 3 / 509	9914 / 0 / 462	2786 / 0 / 156	2383 / 0 / 107	1251 / 0 / 74	1429 / 0 / 100
Goodness-of-fit on F^2	1.057	0.952	1.075	1.066	1.092	1.040
Final R indices [I>2sigma(I)]	R1 = 0.0552, wR2 = 0.1472	R1 = 0.0425, wR2 = 0.1100	R1 = 0.0311, wR2 = 0.0771	R1 = 0.0370, wR2 = 0.1000	R1 = 0.0451, wR2 = 0.1308	R1 = 0.0312, wR2 = 0.0792
R indices (all data)	R1 = 0.0721, wR2 = 0.1638	R1 = 0.0512, wR2 = 0.1155	R1 = 0.0326, R2 = 0.0780	R1 = 0.0435, wR2 = 0.1039	R1 = 0.0508, wR2 = 0.1355	R1 = 0.0331, wR2 = 0.0808
Absolute structure parameter	0.0(2)					
Extinction coefficient	n/a	n/a	n/a	n/a	n/a	n/a
Largest diff. peak and hole [eÅ^{-3}]	0.253 and -0.236	0.486 and -0.345	0.227 and -0.194	0.215 and -0.236	0.216 and -0.216	0.224 d -0.245

Table S3. Torsion angles in **4.1**

Atoms	Torsion angle [°]
C1-C2-O2-C3	-177.68(12)
C2-O2-C3-O3	-2.1(2)
C2-O2-C3-N4	177.91(11)
O2-C3-N4-C4	-177.76(11)
C3-N4-C4-C5	-173.12(12)
O3-C3-N4-C4	2.3(2)
N4-C4-C5-C5[1]	-176.74(14)

[1] -x+2,-y,-z+1

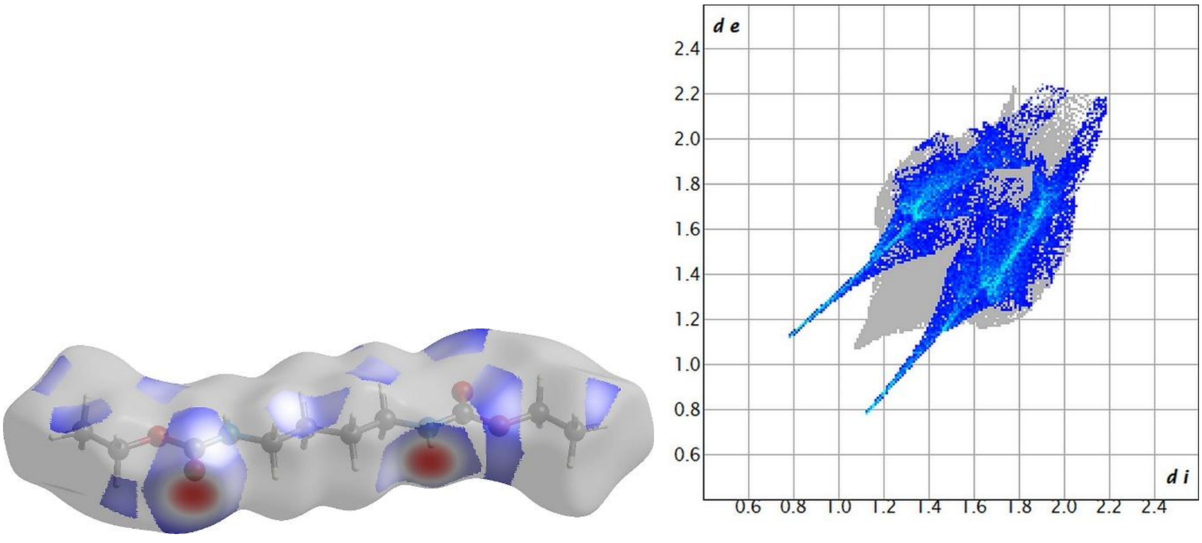

(a)

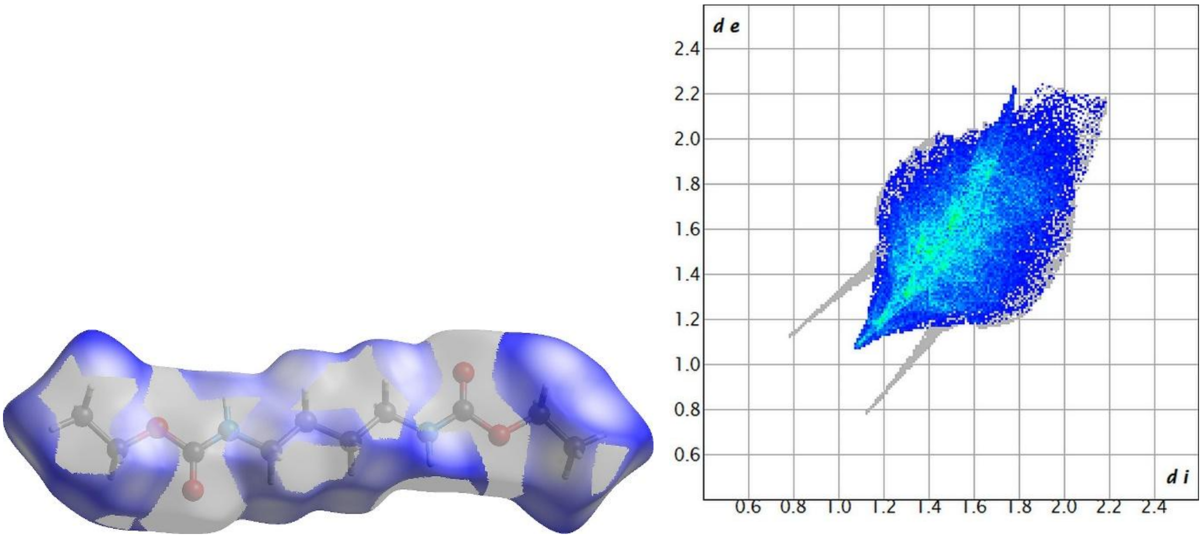

(b)

Figure S1. Hirshfeld surfaces (left) and fingerprints (right) of selected intermolecular interactions found in the crystal network **4.1**: (a) for H O (27.1%), (b) for H H (64.6%). In brackets the surface area is given as a percentage of the total surface area.

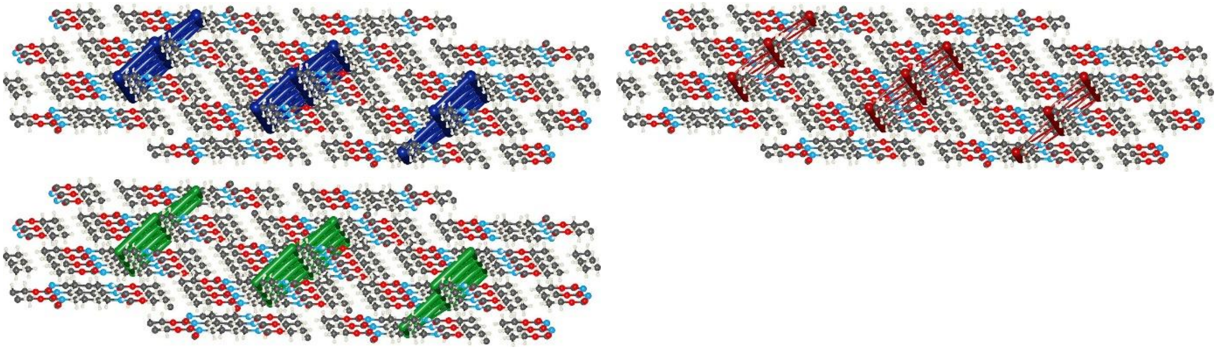

Figure S2. Interactions energy (total in blue, electrostatic in red and dispersion in green) for **4.1**.

Table S4. Torsion angles in **4.2**

Atoms	Torsion angle [°]
C1-C2-O2-C3	156.21(10)
C2-O2-C3-O3	-3.51(16)
C2-O2-C3-N4	176.41(9)
O2-C3-N4-C4	173.47(9)
C3-N4-C4-C5	-110.32(11)
O3-C3-N4-C4	-6.62(18)
N4-C4-C5-C5[1]	65.38(14)

[1] -x+1,-y+1,-z

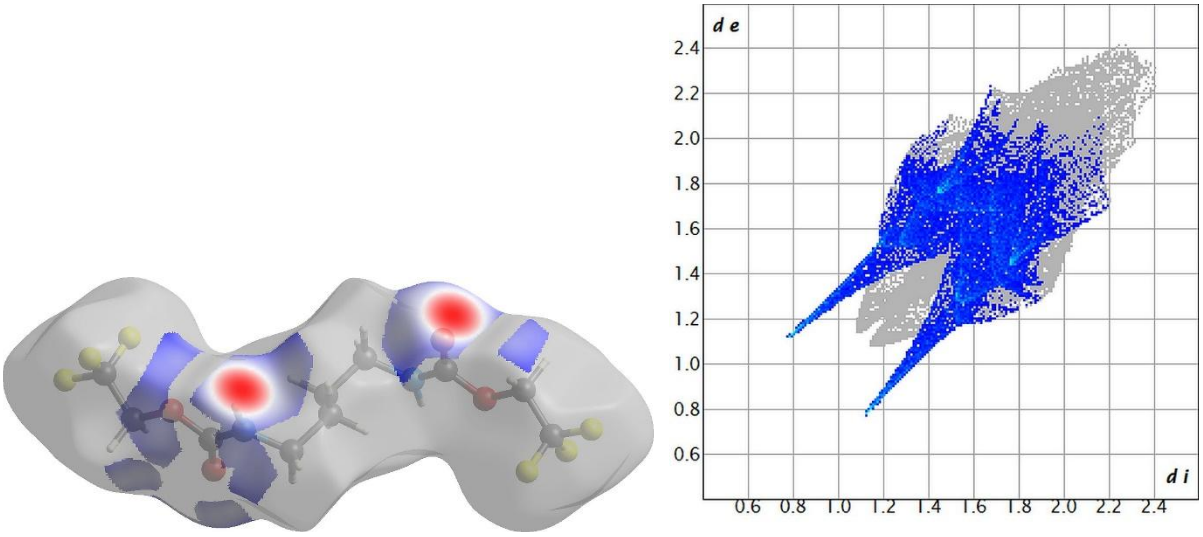

(a)

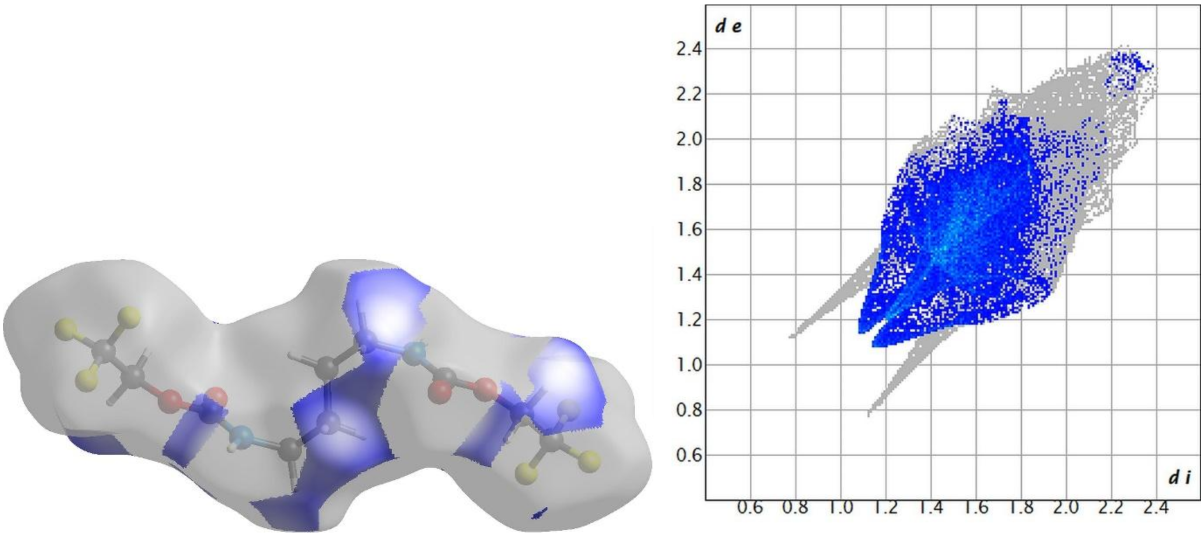

(b)

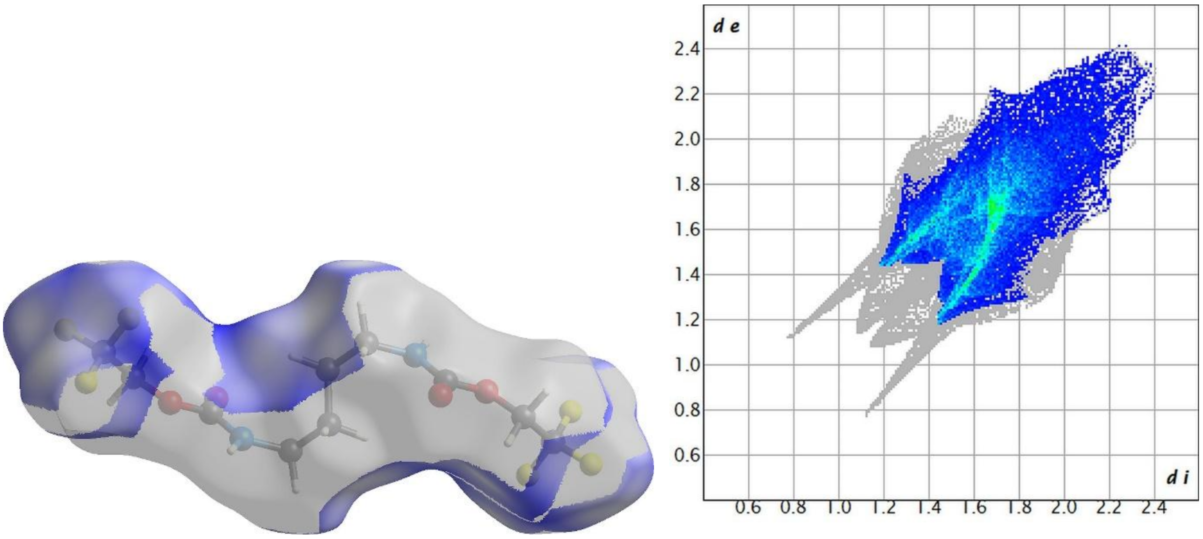

(c)

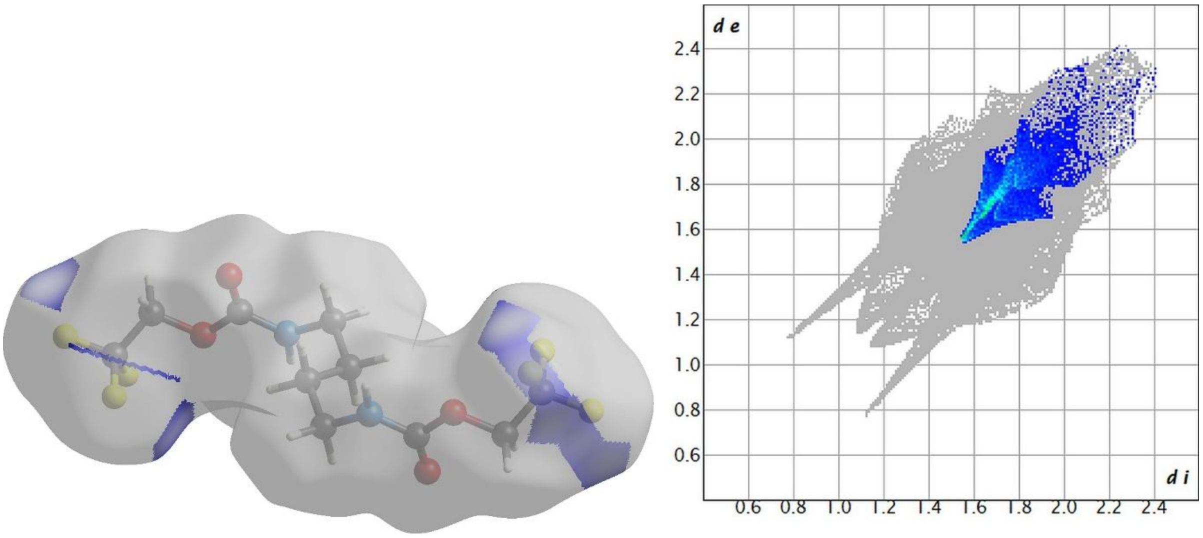

(d)

Figure S3. Hirshfeld surfaces (left) and fingerprints (right) of selected intermolecular interactions found in the crystal network **4.2**: (a) for H O (18.4%), (b) for H H (19.4%), (c)

for H F (45.9%), (d) for F F (9.0%). In brackets the surface area is given as a percentage of the total surface area.

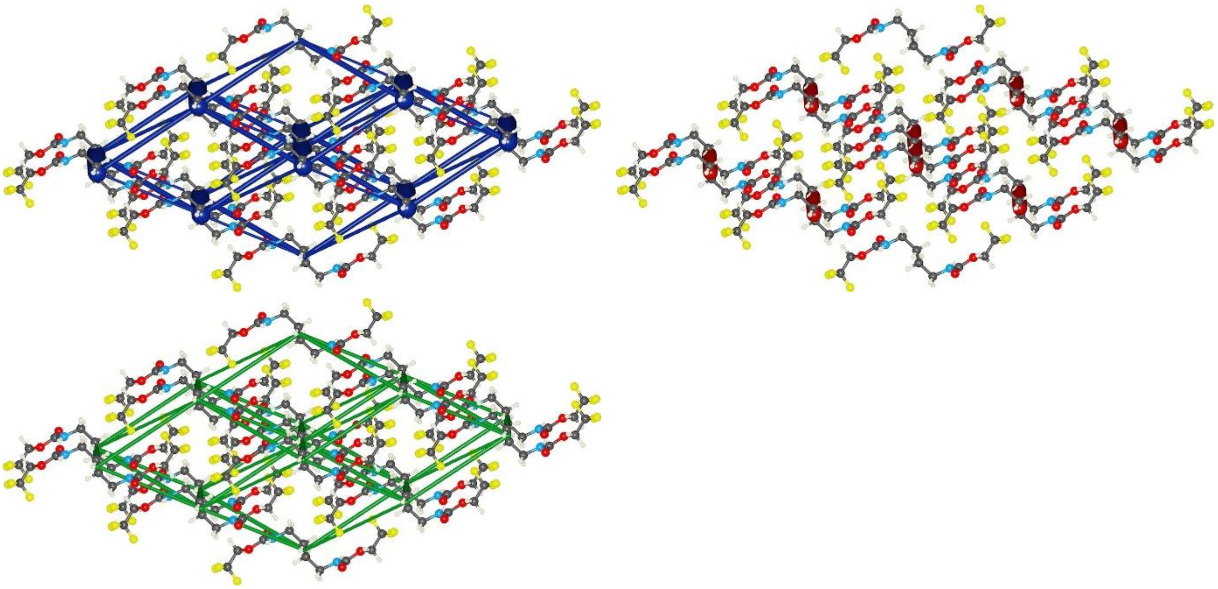

Figure S4. Interactions energy (total in blue, electrostatic in red and dispersion in green) for **4.2**.

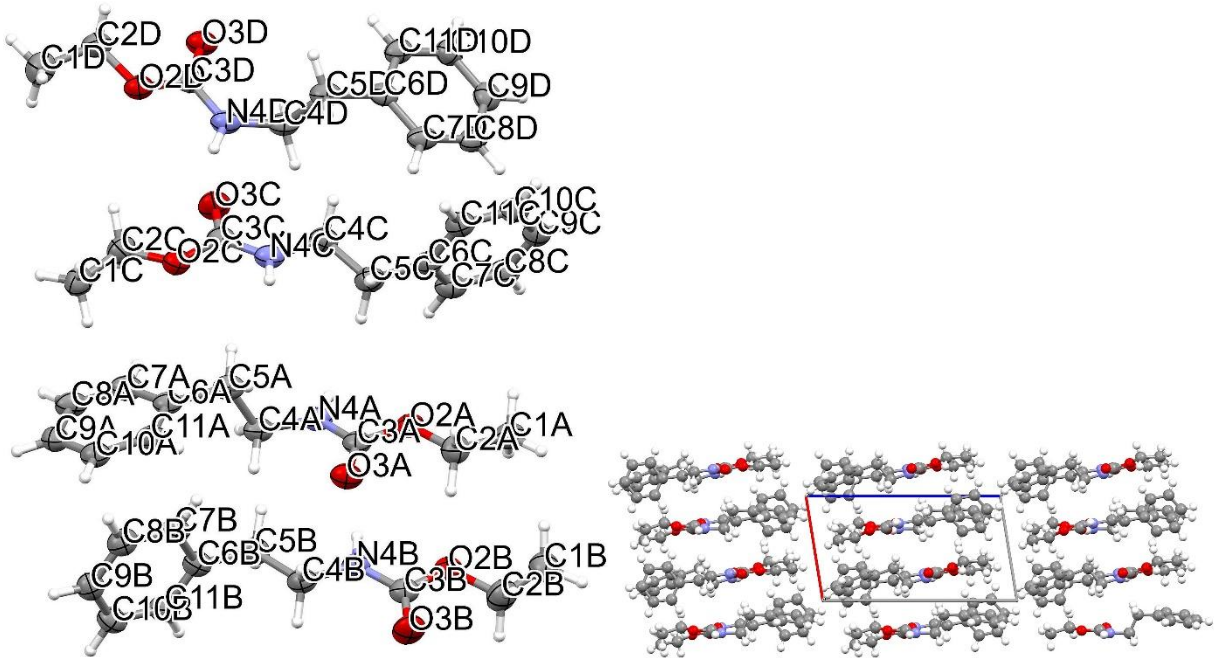

1.1

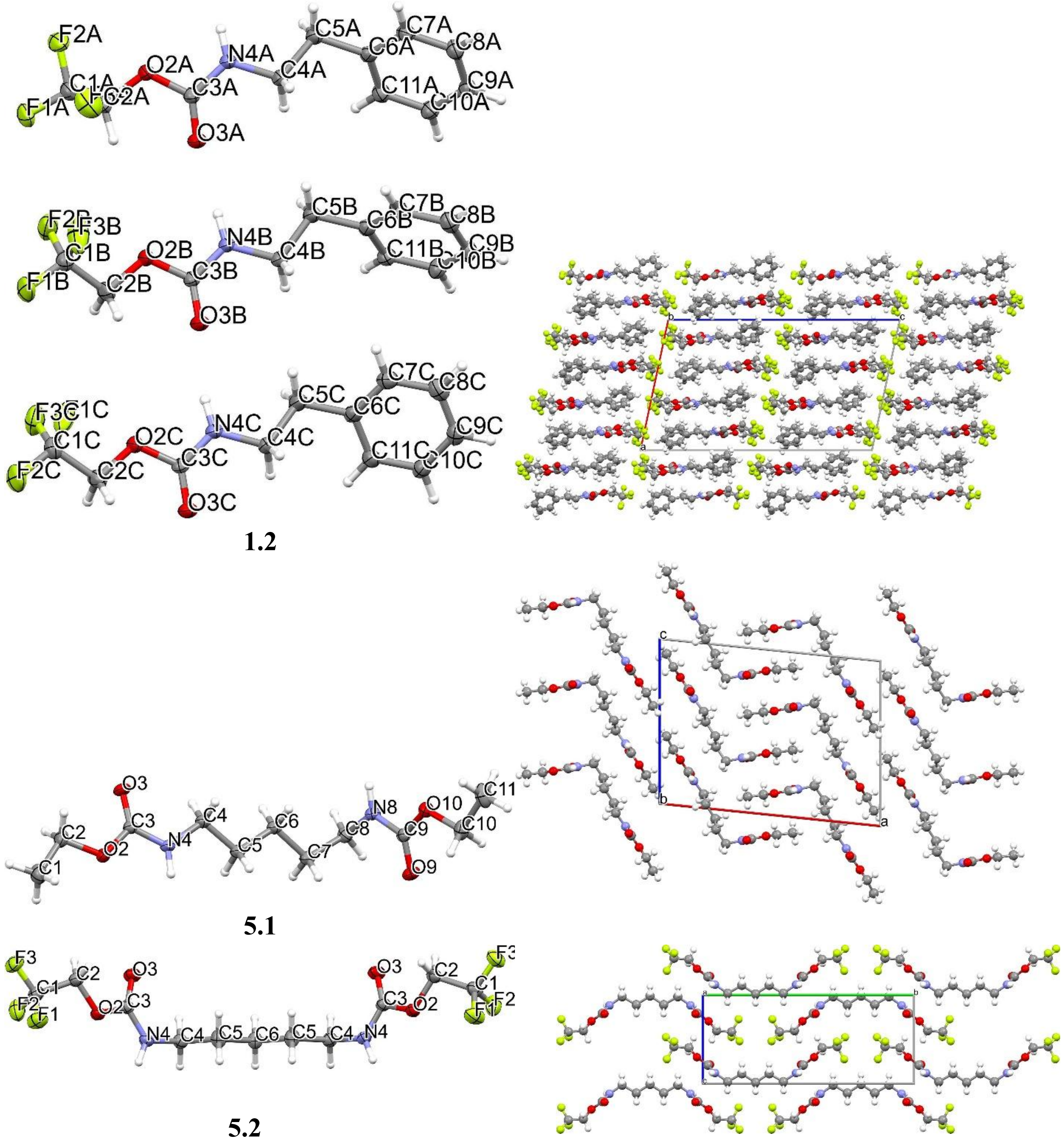

Figure S5. The structure of **1.1**, **1.2**, **5.1**, and **5.2** with ellipsoids at the level of 50% probability and the numbering scheme (left column) and packing motifs (right column).

Selection of synthesis conditions

In preliminary studies, to select an appropriate solvent mixture, we conducted reactions between phenylethylamine (**1**) and ECF in the presence of 1.1 equiv NaOH. We monitored the reaction progress by TLC analysis of the reaction mixture and by the disappearance of phenylethylamine. For mixtures of dioxane, ethyl acetate, methanol, and tetrahydrofuran with water, respectively, TLC analysis indicated incomplete conversion of **1** with ECF after 2 h. In reactions conducted in a mixture of diethyl ether and water, we observed the disappearance of the substrate after 2 h. Reactions of **1**, performed on a 1 mmol scale, in a diethyl ether: water mixture, allowed the isolation of almost quantitative yields of carbamate **1.1** (Table S5). Further reactions of biogenic amines with ECF were carried out in a 1:2 Et_2O: H_2O mixture (see Fig. 3).

Table S5. Selection of solvent composition for the reaction of phenylethylamine with ECF

Solvent mixture	Solvent volume ratio	Yield[a]
Dioxane:water	1 : 1	
Ethyl acetate:water	4 : 1	
Ethyl acetate:water	1 : 1	
Methanol:water	1 : 1	
THF:water	1 : 1	
Diethyl ether:water	1 : 1	95%
Diethyl ether:water	1 : 2	99%
Diethyl ether:water	1 : 4	98%

[a] Isolated yield

For the reaction of fenylethylamine with 2,2,2-trifluoroethyl chloroformate (TFECF) conducted in a diethyl ether – water (1:2) mixture, **1.2** derivative was isolated in 68 % yield.

The reaction was repeated using an ethyl acetate–water (1:2) mixture as the solvent, yielding product **1.2** in 99% yield. The syntheses of the remaining biogenic amine derivatives from TFECF were carried out under such conditions (see Fig. 3).

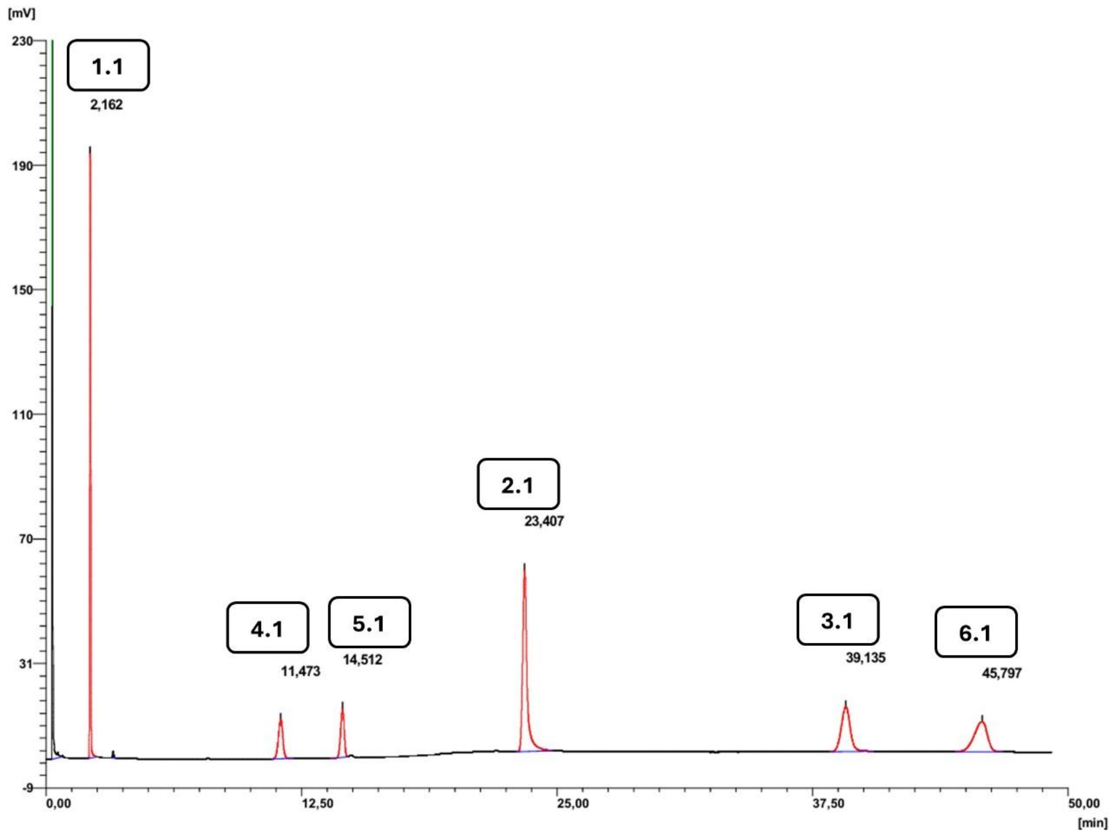

Figure S6. Chromatogram of the mixture of the obtained ECF derivatives of the tested BAs, where: **1.1** - Phen-ECF; **2.1** - Tyr-ECF; **3.1** - Trp-ECF; **4.1** - Put-ECF; **5.1** - Cad-ECF; **6.1** - Spd-ECF

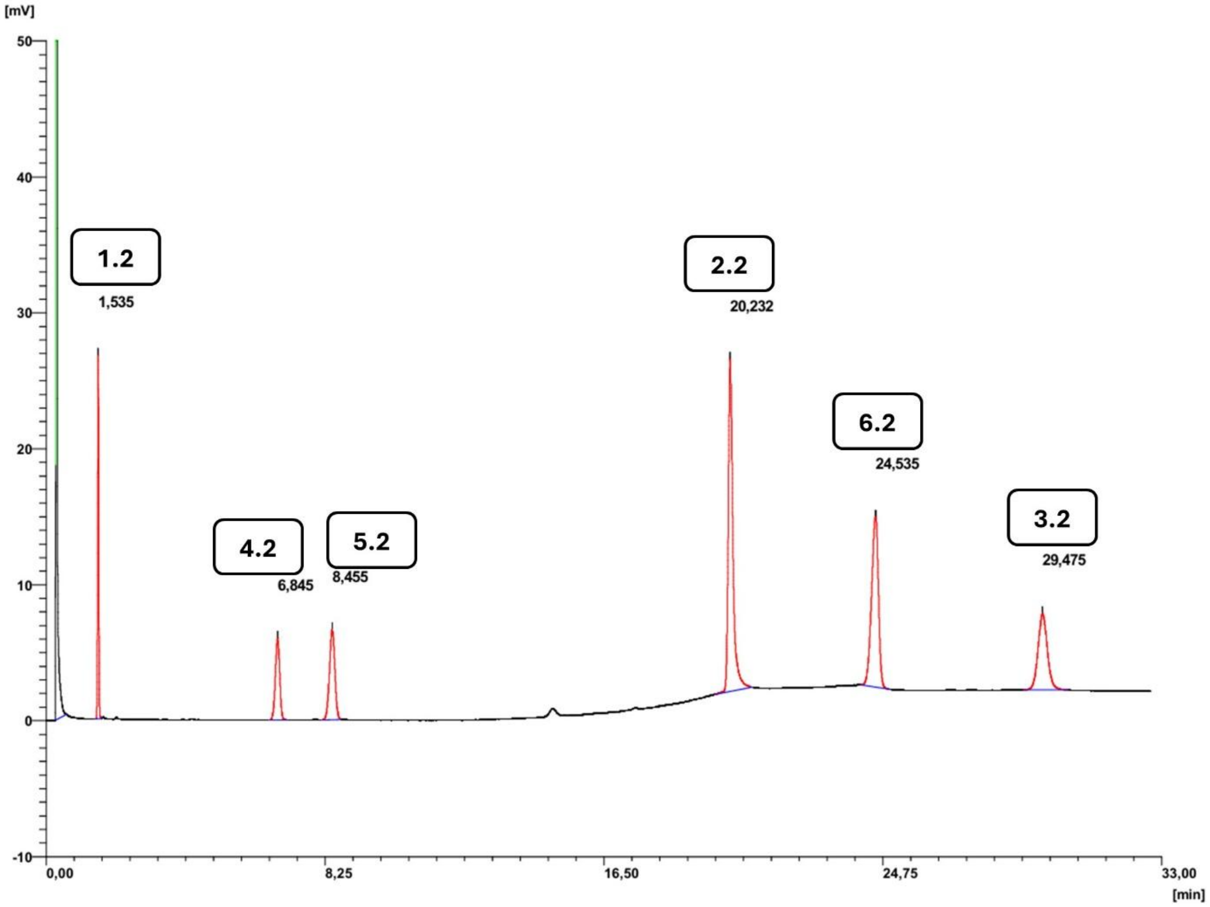

Figure S7. Chromatogram of the mixture of the obtained TECF derivatives of the tested BAs, where: **1.2** - Phen-TFECF; **2.1** - Tyr-TFECF; **3.1** - Trp-TFECF; **4.1** - Put-TFFCF; **5.1** - Cad-TFECF; **6.1** - Spd-TFECF

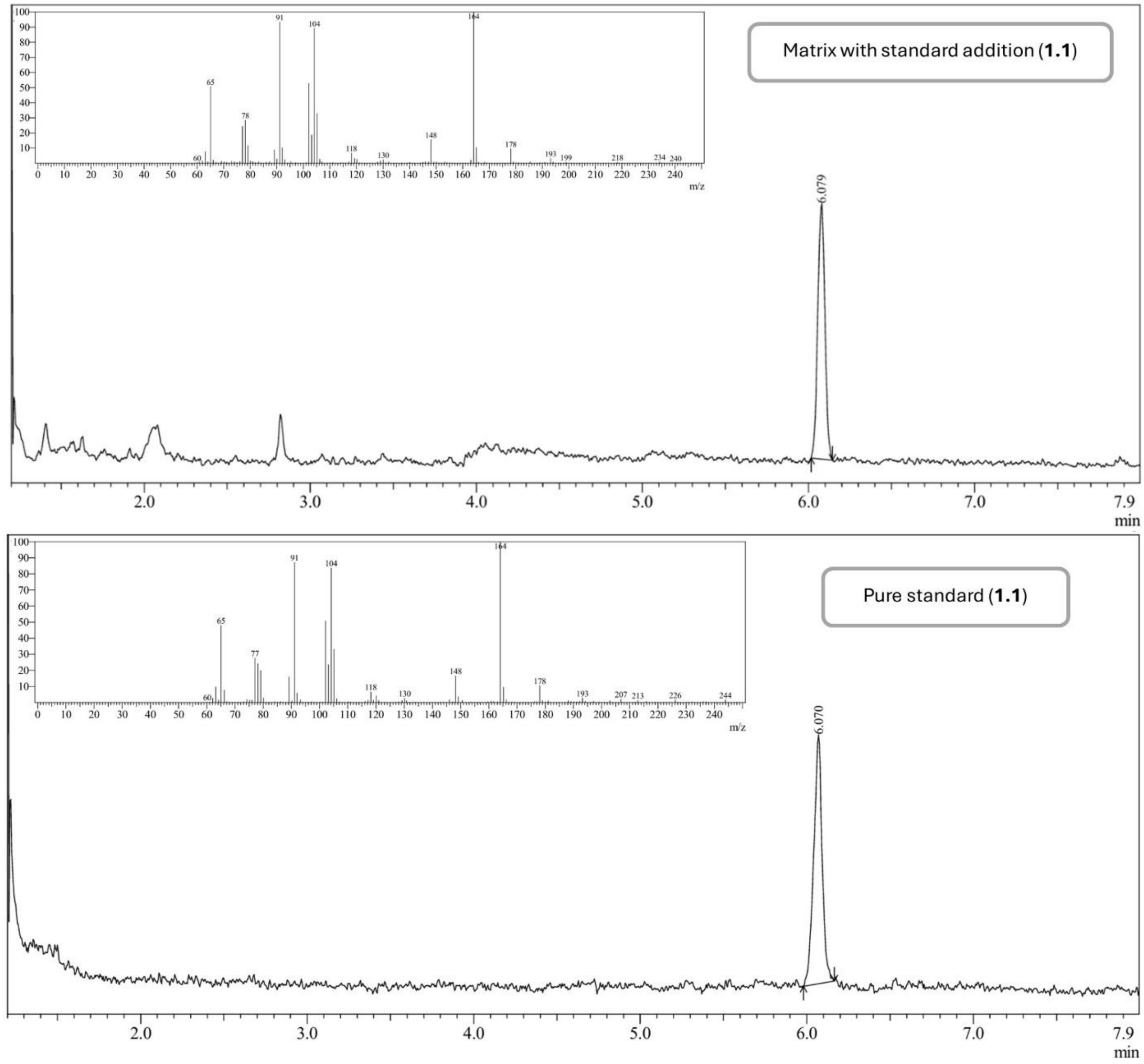

Figure S8. Chromatogram (GC-MS) of the matrix with standard addition (**1.1**) and pure standard (**1.1**)

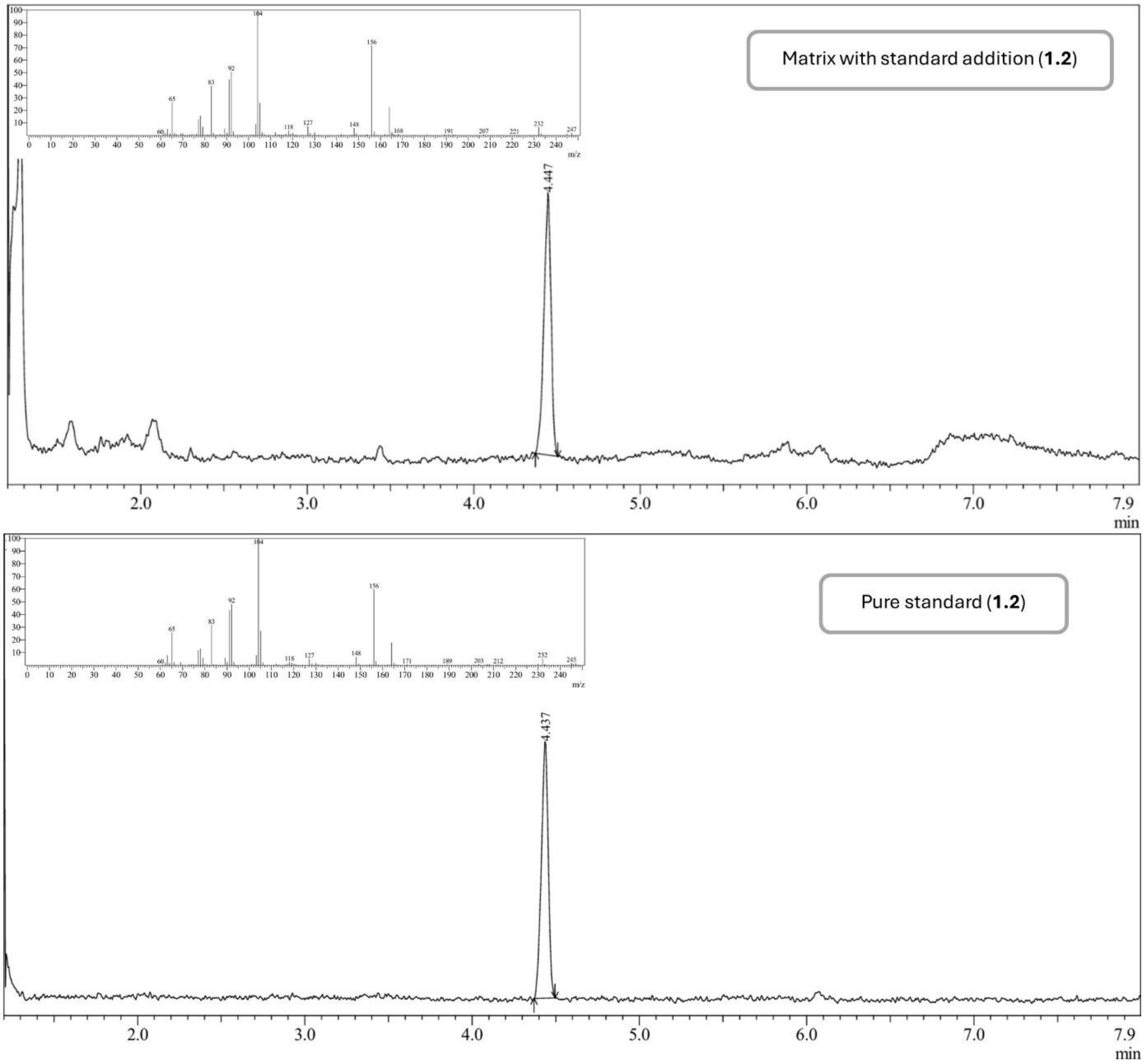

Figure S9. Chromatogram (GC-MS) of the matrix with standard addition (**1.2**) and pure standard (**1.2**)

Table S6. The mean RF and RFF value for the determination of phenylethylamine derivatives (n=7)

	X ± SD	CV [%]
RF (for **1.1**)	103083 ± 5466	5.30
RF (for **2.1**)	134985 ± 1740	1.29
RRF ($RF_{2.1}$/$RF_{1.1}$)	1.31 ± 0.06	4.43

Table S7. Results of two-factor ANOVA for the evaluation of the effect of derivatization reagent on GC–MS analytical results

Source of variation	df	F values	P value
Reagents (A)	1	23.4	< 0.001
Concentration levels (B)	4	13.4	<0.001
A x B	4	0.54	> 0.05
Error	20		
Total	29		

Table S8. Results of fixed-effects two-way ANOVA evaluating the effects of derivatisation reagent and sample type on the analytical response

Source of variation	Sum of squares	df	Mean square	F-value	p-value
Derivatization reagent (A)	0.5436	1	0.5436	19.52	0.00018
Sample (B)	191.776	2	95.8879	3443.0	3.08×10^{-30}
A × B interaction	2.2723	2	1.1362	40.8	1.9×10^{-8}
Within (error)	0.6684	24	0.02785	—	—
**Total**	**195.26**	**29**	—	—	—

Statistical significance was evaluated at $p < 0.05$.

Figure S10. HRMS spectra of the obtained derivatives

Ethyl phenethylcarbamate (**1.1**)

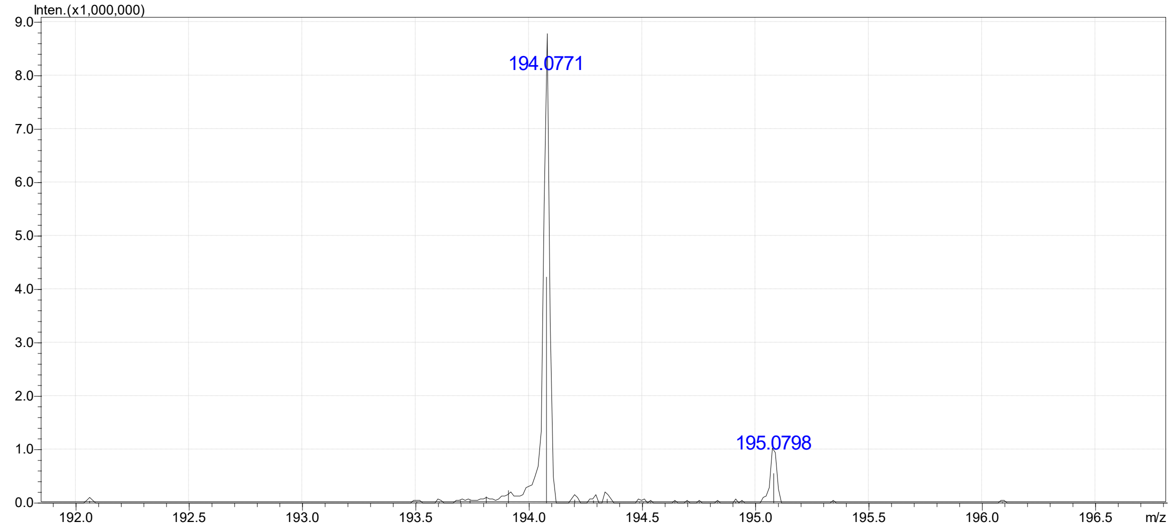

Ethyl (4-hydroxyphenethyl)carbamate (**2.1**)

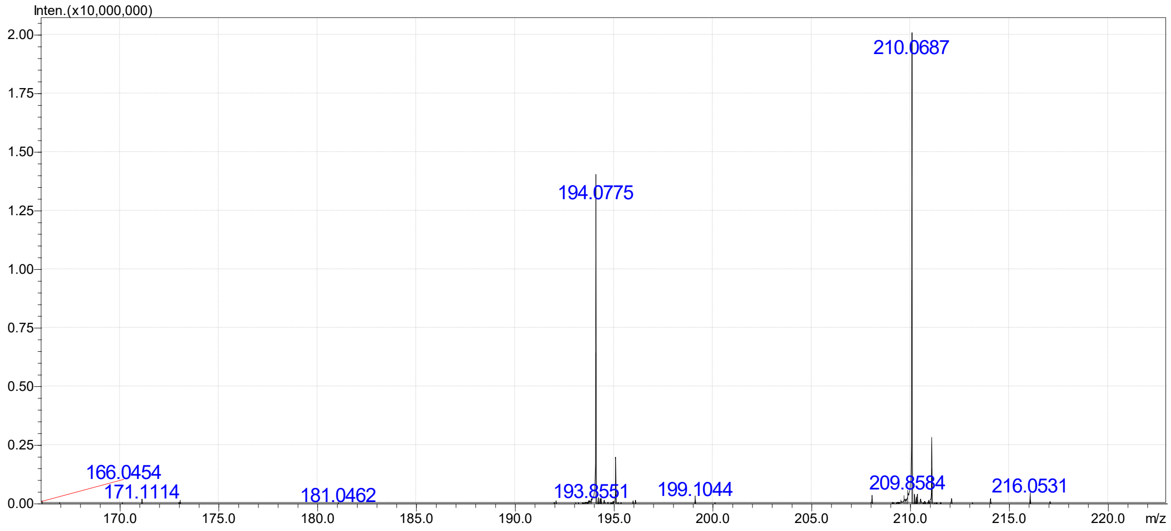

Ethyl (2-(indolin-3-yl)ethyl)carbamate (**3.1**)

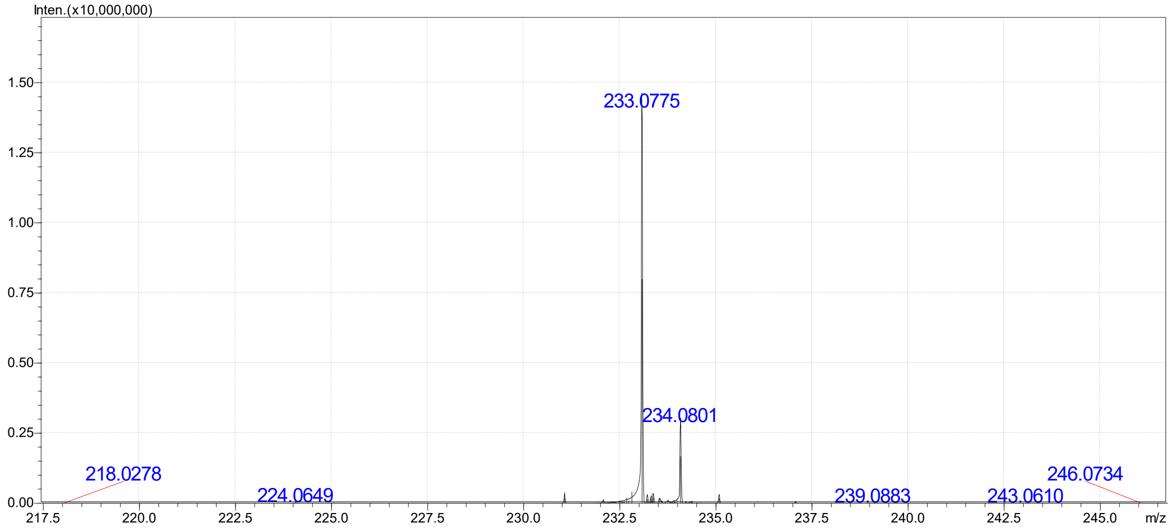

Diethyl butane-1,4-diyldicarbamate (**4.1**)

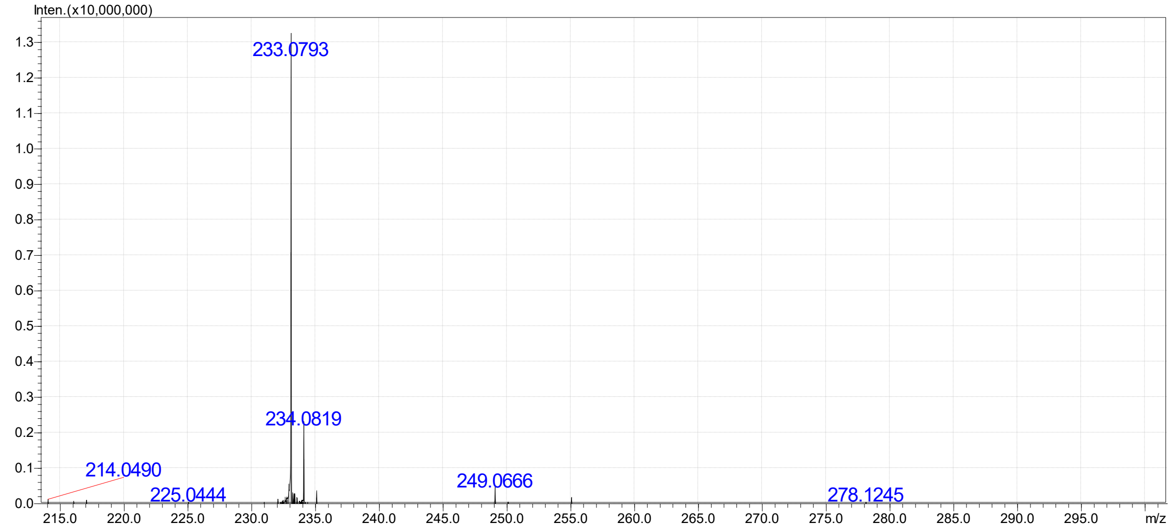

Diethyl pentane-1,5-diyldicarbamate (**5.1**)

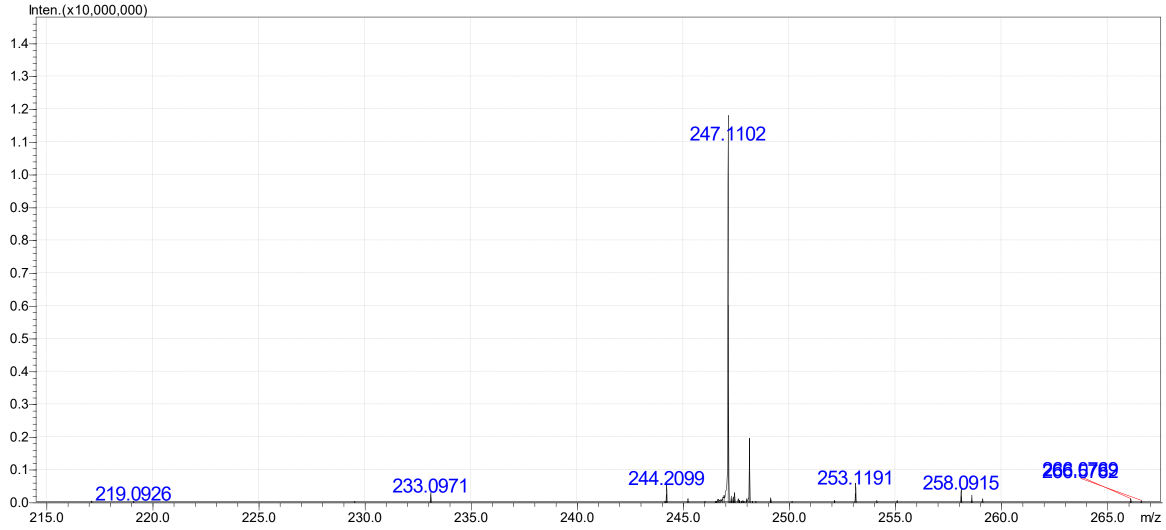

Ethyl (4-((ethoxycarbonyl)amino)butyl)(3-((ethoxycarbonyl)amino)propyl)carbamate (**6.1**)

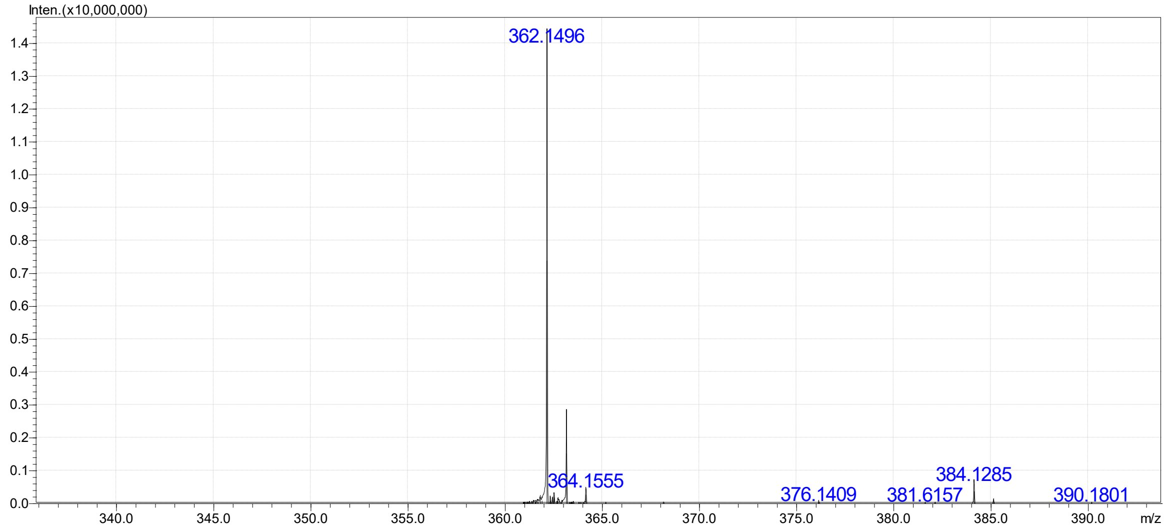

Diethyl butane-1,4-diylbis((3-((ethoxycarbonyl)amino)propyl)carbamate) (**7.1**)

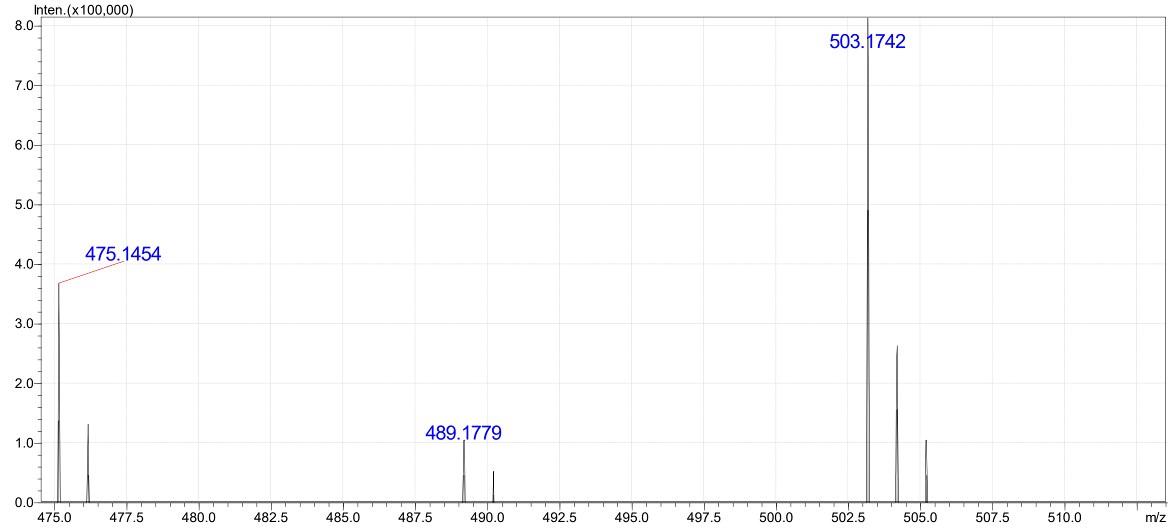

Ethyl (2-(1*H*-imidazol-4-yl)ethyl)carbamate (**8.1**)

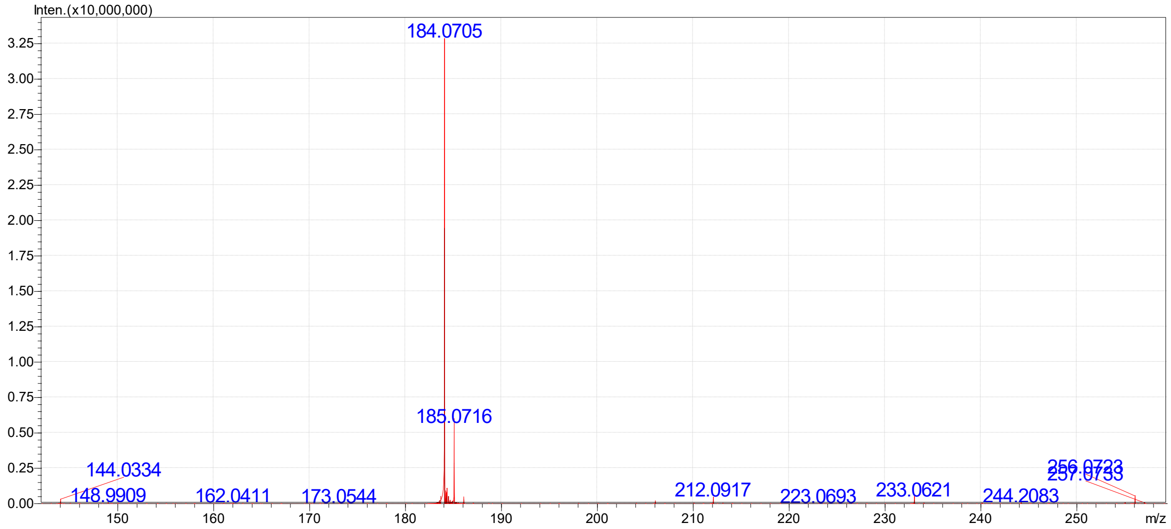

2,2,2-Trifluoroethyl phenethylcarbamate (**1.2**)

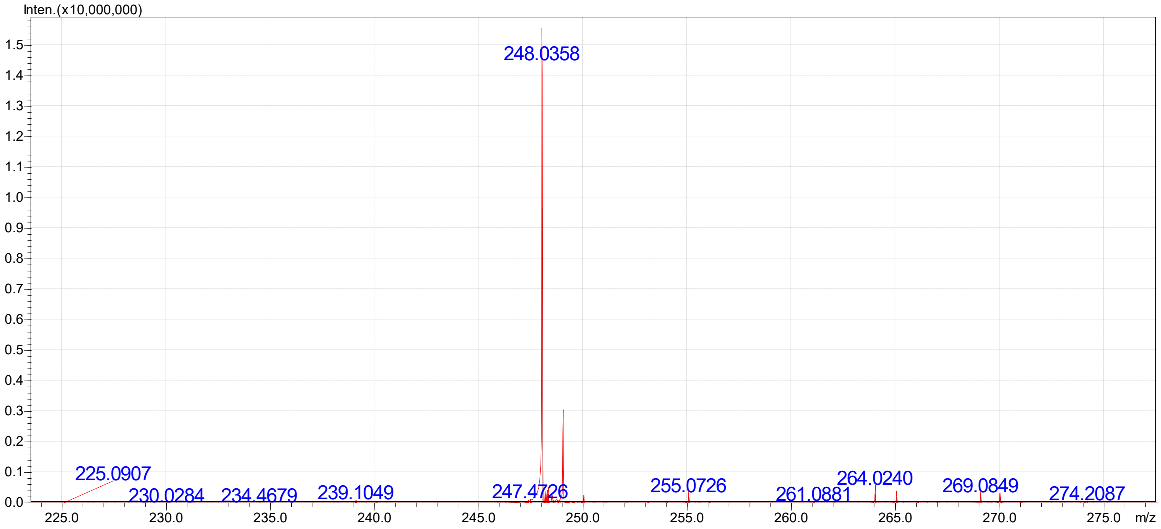

2,2,2-Trifluoroethyl (4-hydroxyphenethyl)carbamate (**2.2**)

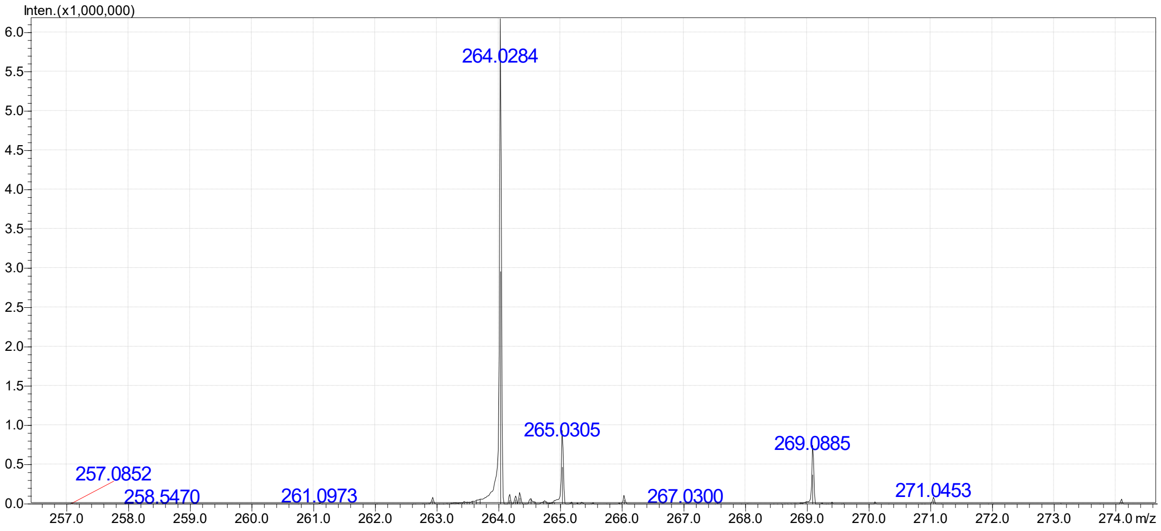

2,2,2-Trifluoroethyl (2-(indolin-3-yl)ethylcarbamate (**3.2**)

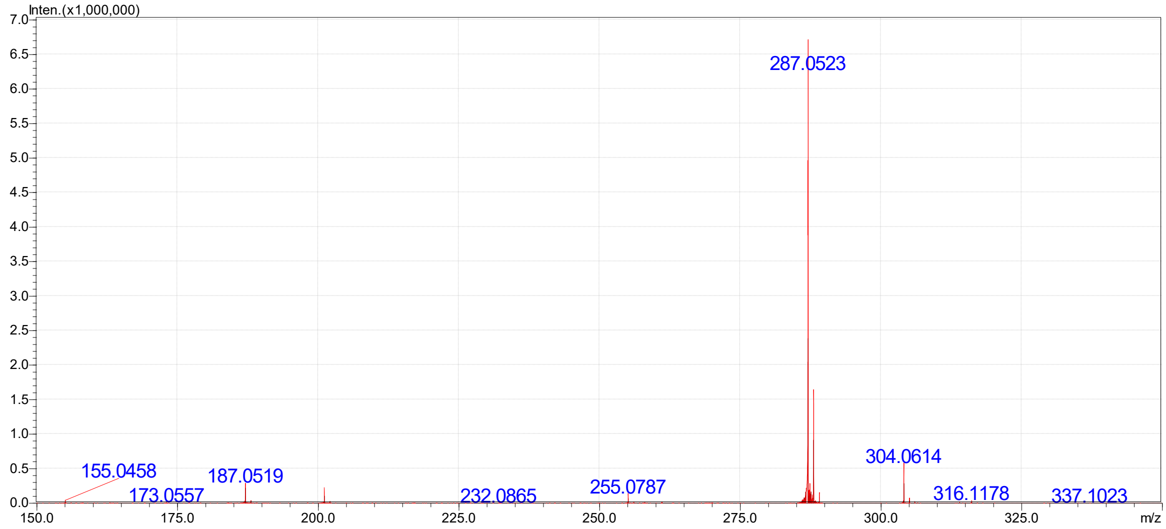

Bis(2,2,2-trifluoroethyl) butane-1,4-diyldicarbamate (**4.2**)

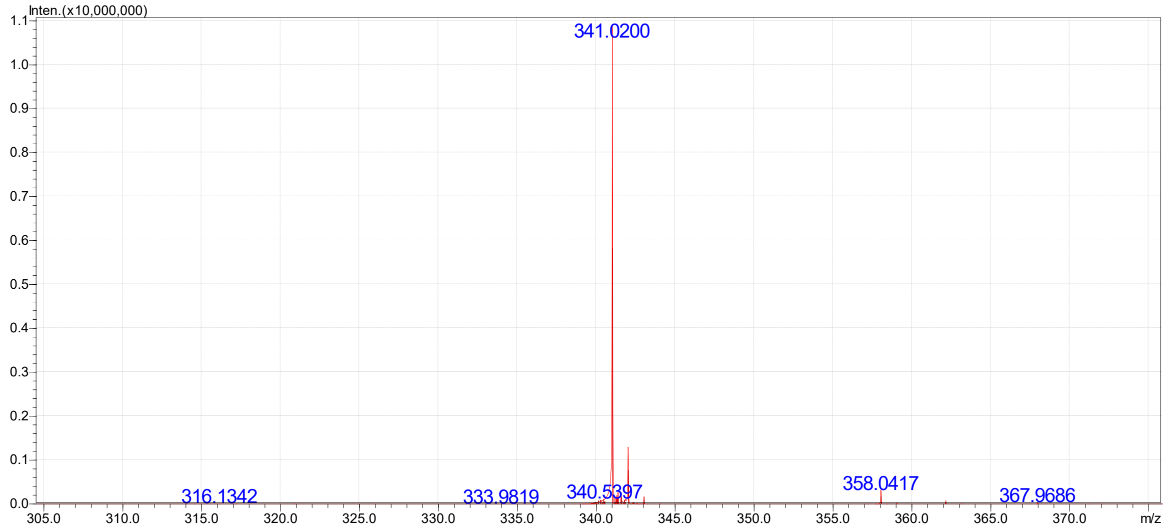

Bis(2,2,2-trifluoroethyl)pentane-1,5-diyldicarbamate (**5.2**)

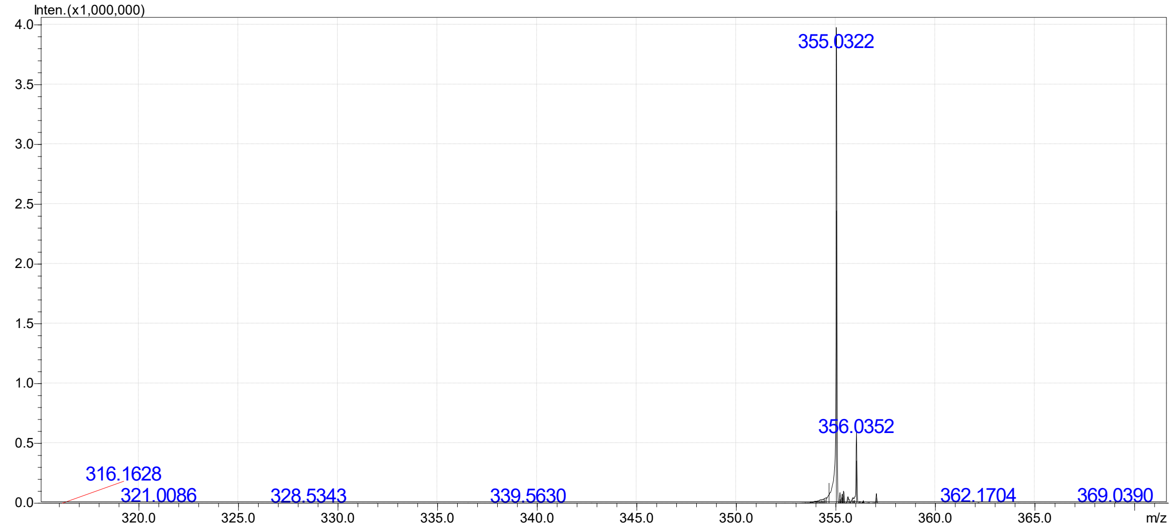

2,2,2-Trifluoroethyl (4-(((2,2,2-trifluoroethoxy)carbonyl)amino)butyl)(3-(((2,2,2-trifluoroethoxy)carbonyl)amino)propyl)carbamate (**6.2**)

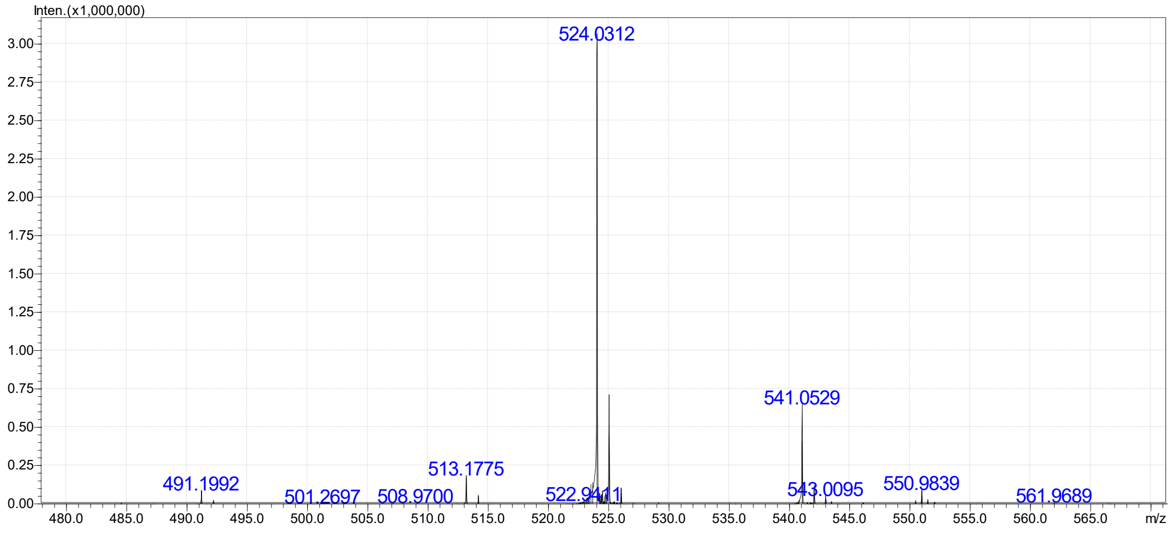

Bis(2,2,2-trifluoroethyl) butane-1,4-diylbis((3-(((2,2,2-trifluoroethoxy)carbonyl)amino) propyl)carbamate) (**7.2**)

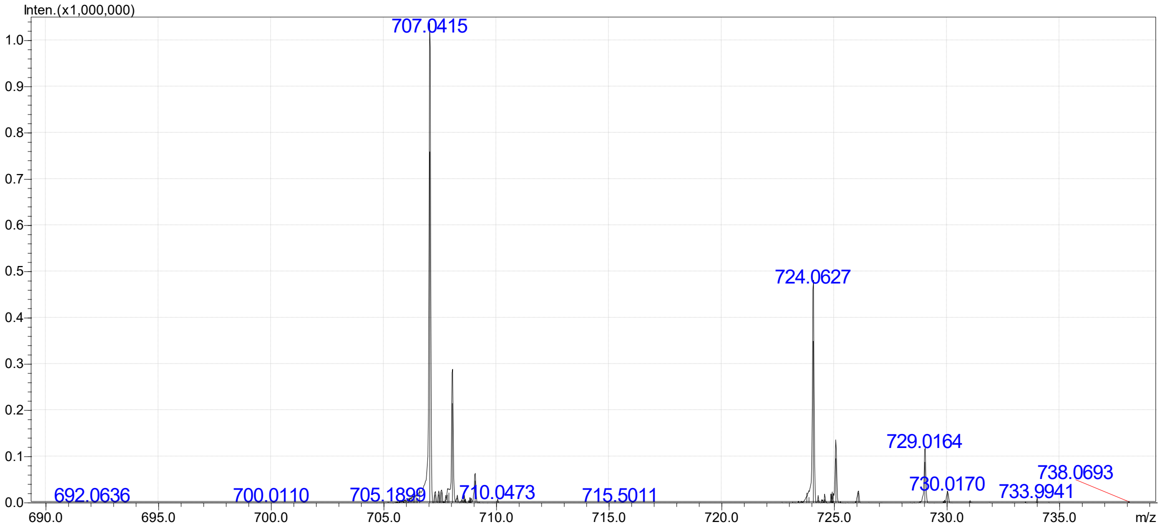

Supplement: Supplementary file 1 [file materials-19-00575-s001.zip › materials-4069627-supplementary.pdf]
